# Energy-efficient integer-only vs. floating-point FLBMF filters for low-power embedded image denoising

**DOI:** 10.1038/s41598-026-51886-0

**Published:** 2026-05-11

**Authors:** Benard Nyangena Kiage, Michael W. Kimwele, Wilson K. Cheruiyot, Jael S. Wekesa

**Affiliations:** 1 Jomo Kenyatta University of Agriculture and Technology, Juja, Kenya; 2 Taita Taveta University, Voi, Kenya

**Keywords:** Integer-only arithmetic, Floating-point computation, Image denoising, Fuzzy logic-based median filter, Embedded medical imaging, Low-power image processing, Fixed-point arithmetic, Real-time signal processing, Mammography, Energy-efficient computing, Engineering, Health care, Mathematics and computing

## Abstract

Fuzzy logic-based median filters (FLBMF) are widely applied in medical imaging to suppress impulse noise while preserving diagnostically relevant structures. Floating-point implementations, although precise, impose substantial computational and memory demands, limiting their deployment on embedded, portable, or low-power imaging systems. This study presents a systematic evaluation of integer-only FLBM and floating-point FLBMF implementations, analyzing trade-offs between denoising performance, computational cost, and hardware efficiency. The integer-only FLBM design leverages quantized membership functions, fixed-point arithmetic, and optimized rule evaluation to reduce arithmetic complexity while maintaining perceptual fidelity, with the floating-point version serving as a precision benchmark. Evaluations were conducted on noisy mammography datasets across multiple noise densities (ρ = 0.3–0.7) using an ARM Cortex-M7 microcontroller and a Xilinx Zynq-7020 FPGA. Performance metrics include image quality (PSNR, SSIM, VIF, FOM), computational efficiency, memory usage, energy consumption, and hardware resource utilization. Experimental results indicate that integer-only FLBMF reduces memory usage by up to 40% and execution time by up to 55% on embedded processors, while maintaining perceptually equivalent denoising quality (ΔPSNR ≤ 0.5dB, ΔSSIM ≤ 0.01). On FPGA platforms, integer-only FLBM implementations achieve lower DSP and LUT utilization, reduced power consumption, and higher operating frequency compared to floating-point versions. Comparisons with classical median, adaptive median, and TV-based filters show that both integer-only FLBM and floating-point FLBMF substantially outperform these baselines. These findings establish integer-only FLBMF as a hardware-friendly, energy-efficient, and clinically faithful solution for real-time, low-power medical imaging, representing the first systematic evaluation of such implementations across MCU and FPGA platforms.

## Introduction

Image denoising is a fundamental preprocessing step in medical imaging, where preserving subtle anatomical structures such as masses and microcalcifications in mammograms is critical for diagnostic reliability. Impulse noise, commonly introduced during low-power acquisition, compression, or wireless transmission, degrades image quality and can obscure clinically relevant features^1–3^. Over the past two decades, nonlinear filters, particularly median-based methods, have been widely used for impulse noise reduction due to their robustness to outliers^4^. Fuzzy logic-based median filters (FLBMF) extend classical median filtering by incorporating fuzzy logic to adaptively discriminate between signal and noise, thereby achieving a better balance between edge preservation and noise suppression^5,6^. Recent studies have shown the effectiveness of FLBMF in enhancing mammographic visibility and supporting the detection of microcalcifications^7,8^.

Despite these advantages, most existing FLBMF designs rely heavily on floating-point arithmetic. Floating-point implementations, while precise, impose substantial computational and memory overhead, limiting their deployment on embedded or portable platforms where energy budgets are tight and floating-point units may be unavailable. As portable medical imaging devices, such as handheld mammography systems and point-of-care diagnostic tools become increasingly important in resource-constrained settings, there is a growing need for denoising algorithms that can operate efficiently on low-power hardware.

Several studies have explored fixed-point and integer-only FLBM realizations for various image processing tasks, demonstrating significant gains in speed, memory, and energy efficiency^11–13^. However, systematic investigations of integer-only FLBMF implementations specifically addressing membership quantization, fixed-point scaling, and division-free blending remain scarce. Moreover, a direct comparison of integer-only FLBM and floating-point FLBMF on both microcontroller (MCU) and field-programmable gate array (FPGA) platforms, with a focus on preserving diagnostic image quality, has not been reported.

To address this gap, *this paper presents a comprehensive implementation-level and system-level comparative evaluation of integer-only* FLBM *and floating-point FLBMF filters for impulse noise removal in mammography images. The focus is on hardware-aware optimization quantization*,* fixed-point scaling*,* and division-free blending*,* enabling deployment on low-power embedded systems*. The main contributions are:

1. Design of an integer-only FLBMF using quantized membership functions, fixed-point arithmetic, lookup-table-based inference, and division-free blending, enabling efficient execution on resource-constrained hardware.
2. Systematic performance evaluation across multiple impulse noise densities (ρ = 0.3, 0.5, 0.7), using image-quality metrics (PSNR, SSIM, VIF, FOM, MAE, EPI) and hardware efficiency metrics (runtime, memory footprint, energy consumption, logic utilization).
3. Dual-platform validation on an ARM Cortex-M7 microcontroller (NXP i.MX RT1170) and a Xilinx Zynq-7020 FPGA, demonstrating that the integer-only FLBM design preserves perceptual quality (ΔPSNR ≤ 0.7dB, ΔSSIM ≤ 0.01) while achieving substantial reductions in execution time (≈ 30–55%), energy (≈ 79% on MCU), and logic resources (≈ 40–60% on FPGA).
4. Benchmarking against classical methods (median, adaptive median, TV-based) to highlight the advantage of fuzzy adaptation, and analysis of the trade-offs between 12-bit and 16-bit fixed-point precision on FPGA.

The remainder of this paper is organized as follows. Section 2 reviews related work on fuzzy-median filters, fixed-point hardware implementations, and embedded denoising strategies. Section 3 describes the FLBMF filter mechanics, the integer-only FLBM realization, and the experimental setup, including datasets, noise models, evaluation metrics, and hardware platforms. Section 4 presents and discusses the experimental results, covering qualitative visualization, quantitative performance across noise levels, per-image analysis, extended metrics, comparison with state-of-the-art methods, and hardware efficiency evaluation. Finally, Sect. 5 concludes the paper and outlines directions for future work.

## Related work

Image denoising is a critical preprocessing step in medical imaging, where preserving fine structural details under noise corruption directly affects diagnostic accuracy. In mammography, linear filtering techniques such as mean and Wiener filters suppress random noise but blur high-frequency structures. Nonlinear approaches, particularly median filtering, better handle impulse noise but struggle to preserve minute microcalcifications and often degrade important textures at high noise levels. Enhanced variants including adaptive median, bilateral filtering, and total variation minimization offer improved edge preservation but incur substantial computational cost, limiting their suitability for real-time, portable imaging systems. These limitations have motivated the development of fuzzy logic based denoising approaches, which introduce context-sensitive decision mechanisms to better balance noise suppression and detail preservation.

The present study builds on these developments, focusing specifically on resource-efficient fuzzy realizations suitable for constrained diagnostic platforms. Two converging trends are evident in the literature: increasingly sophisticated fuzzy-median filters capable of preserving diagnostic details under high noise conditions, and widespread adoption of fixed-point arithmetic in embedded image and signal processing to reduce energy consumption and hardware costs. However, these trends have not yet intersected, as fuzzy logic-based median filters (FLBMF) have not been systematically studied under integer-only FLBM constraints, particularly in relation to membership quantization and rule evaluation. This gap motivates the present study, which unifies both computational and algorithmic perspectives.

### Fuzzy logic-based median filters for image denoising

Classical median filtering is widely used for impulse noise removal due to its robustness against outliers, but it treats all pixels equally, often blurring fine textures or diagnostically relevant details^4^. To overcome this limitation, fuzzy logic based median filters (FLBMF) integrate fuzzy inference to adaptively discriminate between noisy and signal pixels, thereby improving edge preservation^5,6^. The general structure includes fuzzification of local intensity differences, rule based inference, and defuzzification to compute a weighted combination of the original pixel and the neighborhood median. Variants such as adaptive fuzzy weighted median filters and hybrid FLBMF Selective Median TV schemes^7^ have demonstrated superior performance in mammogram denoising, especially under extreme impulse noise. However, these implementations typically rely on floating point arithmetic, which imposes high computational and memory demands, limiting their use in low power embedded systems.

Recent work has continued to advance fuzzy logic based median and hybrid filters for impulse noise removal. Singh^8^ proposed the AT-2FF filter, a type-2 fuzzy weighted mean method with adaptive thresholding, which demonstrates strong resilience to salt and pepper noise while maintaining structural fidelity. Likewise, Das et al.^9^ introduced an adaptive fuzzy filter incorporating convolved feature vectors, reflecting a broader trend toward feature aware fuzzy frameworks. In such designs, convolutional kernels extract local descriptors that are classified using fuzzy inference rules prior to selective denoising. Beyond algorithmic innovations, implementation efficiency has also gained attention. Ben Atitallah et al.^10^ presented a vector median rational hybrid filter optimized via HW/SW co-design for real time color image denoising, highlighting the relevance of hardware aware design. However, these designs remain floating point based.

### Fixed-point and integer-only flbm implementations in image processing

To address the resource constraints of embedded platforms, many image processing algorithms have been adapted to fixed-point or integer-only FLBM arithmetic. Two complementary directions have emerged: FPGA-centric hardware implementations and microcontroller (MCU) -oriented software strategies.

***FPGA Implementations;*** Most implementation-focused studies in the denoising literature evaluate FPGA and ASIC realizations, where fixed-point arithmetic predominates due to strict constraints on area, power, and throughput. Across these works, carefully selected word lengths consistently demonstrate that high filtering quality can be maintained while achieving substantial hardware savings. For example, Varghese^11^ showed that 12–14-bit fixed-point precision was sufficient for generic risk-estimation denoising, yielding less than 0.3dB PSNR loss relative to a 32-bit floating-point baseline while reducing LUT usage by nearly half. Similarly, Bundschuh et al.^12^ reported a real-time FPGA-based median filtering architecture that operated efficiently on 1080p video streams using only 15k LUTs and 8 DSP slices when restricted to 10–12-bit data-paths. General analyses of DSP workloads further reinforce the role of bit-width optimization in hardware efficiency. Li et al.^13^ demonstrated that fixed-point tuning in FFT accumulation stages (12–16 bits) could reduce energy consumption by up to 38% with minimal accuracy degradation. Hardware-aware designs emphasize additional optimization strategies such as quantized lookup tables, pipelined data-paths, and reciprocal-multiply normalization that significantly reduce logic utilization while eliminating high-cost division operations^14^.

***MCU and Software-Oriented Strategies;*** In scenarios where floating-point units (FPUs) are unavailable or energy budgets are limited, integer-only FLBM and fixed-point implementations have shown considerable promise at the microcontroller and software levels. Using 16-bit fixed-point arithmetic, Bilal and Janjua^15^ demonstrated smartphone-based DSP pipelines that reduced latency by up to 45% compared to floating-point implementations while consuming approximately 30% less memory. Similarly, Zoni et al.^16^ analyzed fixed-point extensions on RISC-V architectures and found that 16-bit add/multiply operations were particularly attractive for MCU-class devices, consuming 40–50% less energy than equivalent single-precision floating-point instructions. In MRI reconstruction applications, Takeda et al.^17^ showed that 14–16-bit fixed-point precision was sufficient for real-time throughput without introducing noticeable artifacts. More recent work by Spagnolo et al.^18^ implemented an energy-efficient bilateral filter using fixed-point arithmetic for medical imaging, achieving substantial hardware savings.

Despite these advances, none of these studies systematically address the quantization of fuzzy logic-based median filters, nor do they provide a direct comparison of integer-only FLBM and floating-point FLBMF across both MCU and FPGA platforms with full quality metrics.

### Comparative analysis of quantization methods for denoising

Various quantization strategies have been proposed to balance numerical accuracy and hardware efficiency. Uniform quantization is most common due to its simplicity, while non-uniform and adaptive quantization schemes can better preserve perceptual details in low-intensity regions^19^. Recent work has applied deep learning to image denoising, where convolutional neural networks (CNNs) have demonstrated strong performance^20^, and quantized implementations have been explored to improve hardware efficiency on platforms such as FPGAs^21^. However, such methods remain computationally intensive and rely on large training datasets, making them less suitable for ultra-low-power embedded medical devices. For traditional filters, comparative studies of quantization schemes are scarce. The present work adopts a uniform quantization approach with carefully chosen word lengths and a division-free blending strategy, and it benchmarks the integer-only FLBMF against both its floating-point counterpart and classical denoising methods, thereby filling a gap in the literature.

### Summary and research gap

In summary, while fuzzy logic-based median filters are well established for impulse noise removal, their floating-point nature hinders deployment on resource-constrained embedded platforms. At the same time, fixed-point implementations have been widely adopted for various image processing tasks, demonstrating significant improvements in speed, memory, and energy. However, a systematic evaluation of integer-only FLBMF, including membership quantization, fixed-point scaling, and division-free blending has not been reported, nor has a direct comparison between integer-only FLBM and floating-point FLBMF across both MCU and FPGA platforms been performed. The present work addresses these gaps by providing a comprehensive comparative evaluation, demonstrating that integer-only FLBMF achieves near-identical diagnostic quality while delivering substantial hardware efficiency gains.

## Methodology

**Fig. 1:**
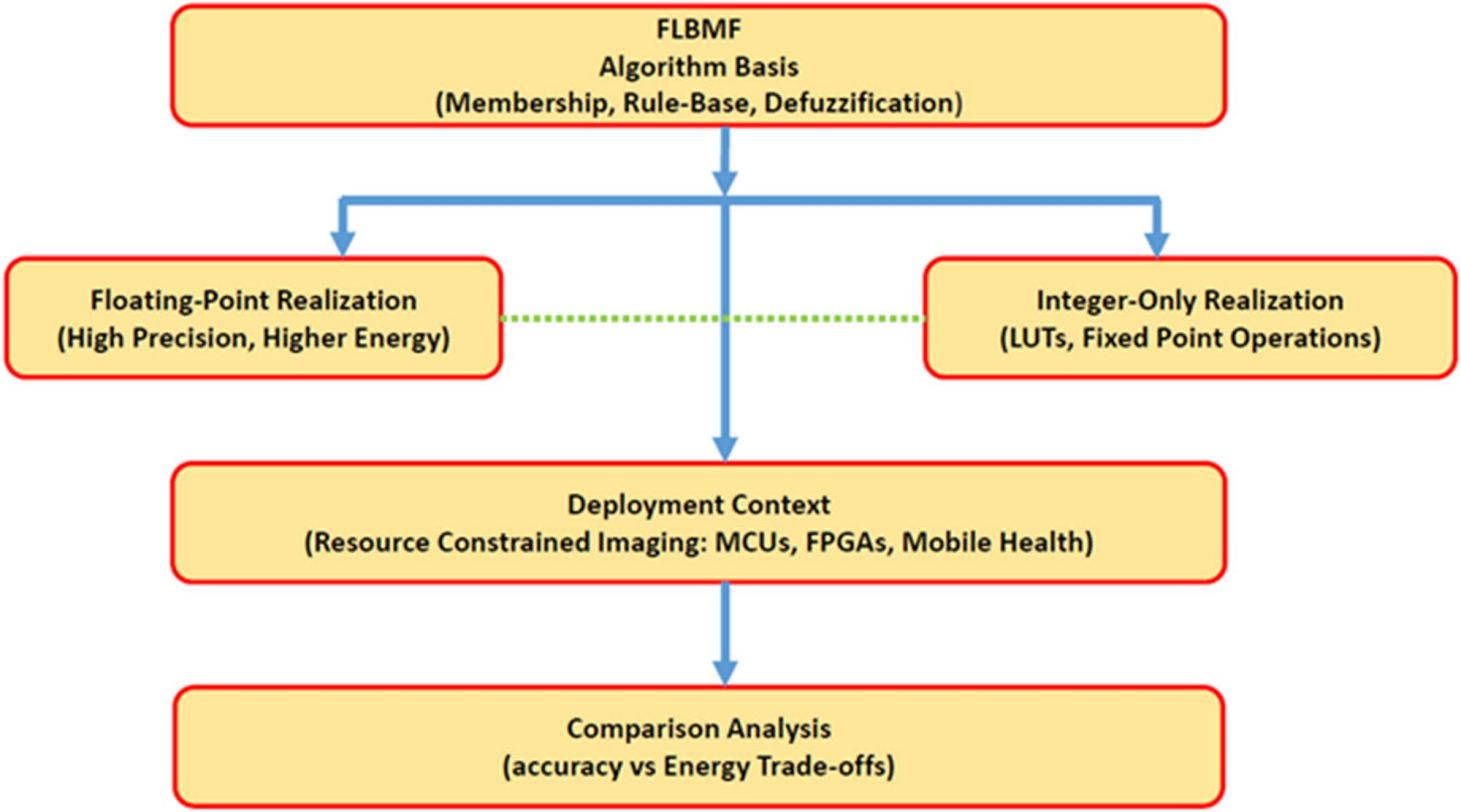
illustrates the conceptual framework of the proposed methodology.

This section describes the proposed FLBMF filtering process, its integer-only FLBM realization, and the experimental protocol used to evaluate both accuracy and resource efficiency. The methodology is organized as a tiered framework connecting deployment context, arithmetic realization, algorithmic design, and performance assessment. Fundamentally, the Fuzzy Logic-Based Median (FLBMF) filter integrates fuzzy membership evaluation, rule inference, and defuzzification to suppress impulse noise in mammogram images. This study focuses on the implementation-level and system-level optimization of the FLBMF, specifically the integer-only FLBM realization, quantization strategies, fixed-point scaling, and division-free blending rather than on introducing a fundamentally new denoising algorithm. Building on this, the framework systematically compares two realizations; floating-point and integer-only FLBM and evaluates their suitability for low-power diagnostic imaging platforms, focusing on denoising accuracy, computational efficiency, and memory footprint. This methodology and the relationships between its main elements are depicted in Fig. 1.

The Fig. 1, presents the Conceptual framework of the FLBMF approach, illustrating the relationship between algorithmic basis, implementation strategies (floating-point and integer-only) FLBM, deployment context, and performance evaluation in terms of accuracy-energy trade-offs. The upper tier depicts the core FLBMF operations: membership evaluation, fuzzy rule inference, and defuzzification. The lower tier contrasts the two arithmetic realizations targeting FPGA and MCU platforms.

### FLBMF filter mechanics

The Fuzzy Logic-Based Median (FLBMF) filter enhances the ability to distinguish between signal and noisy pixels by incorporating fuzzy inference into the decision-making process. The FLBMF framework operates in three main phases: window selection and median computation, difference evaluation and fuzzification, and rule evaluation with blending.

***1. Window Selection and Median Computation***: For each target pixel $\:{x}_{i,j}$ a sliding window W, k×k is extracted. The median $\:m$ of this window is computed as:

1 $$\:m=median\left\{{x}_{p,q}\left|(p,q)\right.\in\:W\right\}.$$

Where, $\:{x}_{p,q}$ is the intensity value of a pixel at position$\:(p,q)$, $\:W$ is the neighborhood around the central pixel (i, j), e.g., a 3 × 3 or 5 × 5). The median $\:m$ serves as the primary noise-suppressed candidate value.

***2. Difference Evaluation and Fuzzification***: The intensity difference between the central pixel and each neighbor is calculated:

2 $$\:{d}_{p,q}=\left|{x}_{p,q}-{x}_{i,j}\right|,\:\left(p,q\right)\in\:W.$$

Where: $\:{x}_{i,j}$ is the central pixel at coordinates (i, j), $\:{x}_{p,q}$ is the neighboring pixel located at coordinates (p, q), W is the neighborhood window around the central pixel (for example, a 3 × 3 or 5 × 5 block of pixels), $\:\mid\:\cdot\:\mid\:$ is the absolute value, ensuring the difference is always non-negative, $\:{d}_{p,q}$ is the intensity difference between the central pixel and its neighbor. To measure the probability that a pixel is noise, each difference is mapped to a fuzzy membership function $\:\mu\:\left(d\right)$, which is usually triangular or Gaussian in shape. The following is a typical parameterization:

3 $$\:{\mu\:}_{noise}\left(d\right)=\left\{\begin{array}{c}1\:\:\:\:\:\:\:\:\:\:\:\:\:\:\:\:\:\:\:\:\:\:\:\:\:\:\:\:\:\:\:\:d\ge\:{T}_{max,}\:\:\:\:\:\:\:\:\:\:\:\:\:\:\:\\\:\frac{d-{T}_{min}}{{T}_{max}-{T}_{min}}\:\:\:\:\:\:\:\:\:\:\:{T}_{min}<d<{T}_{max,}\\\:0\:\:\:\:\:\:\:\:\:\:\:\:\:\:\:\:\:\:\:\:\:\:\:\:\:\:\:\:\:\:\:\:\:d\le\:{T}_{min,\:\:\:\:\:\:\:\:\:\:\:\:\:\:\:\:\:\:\:\:}\end{array}\right.$$

**Fig. 2:**
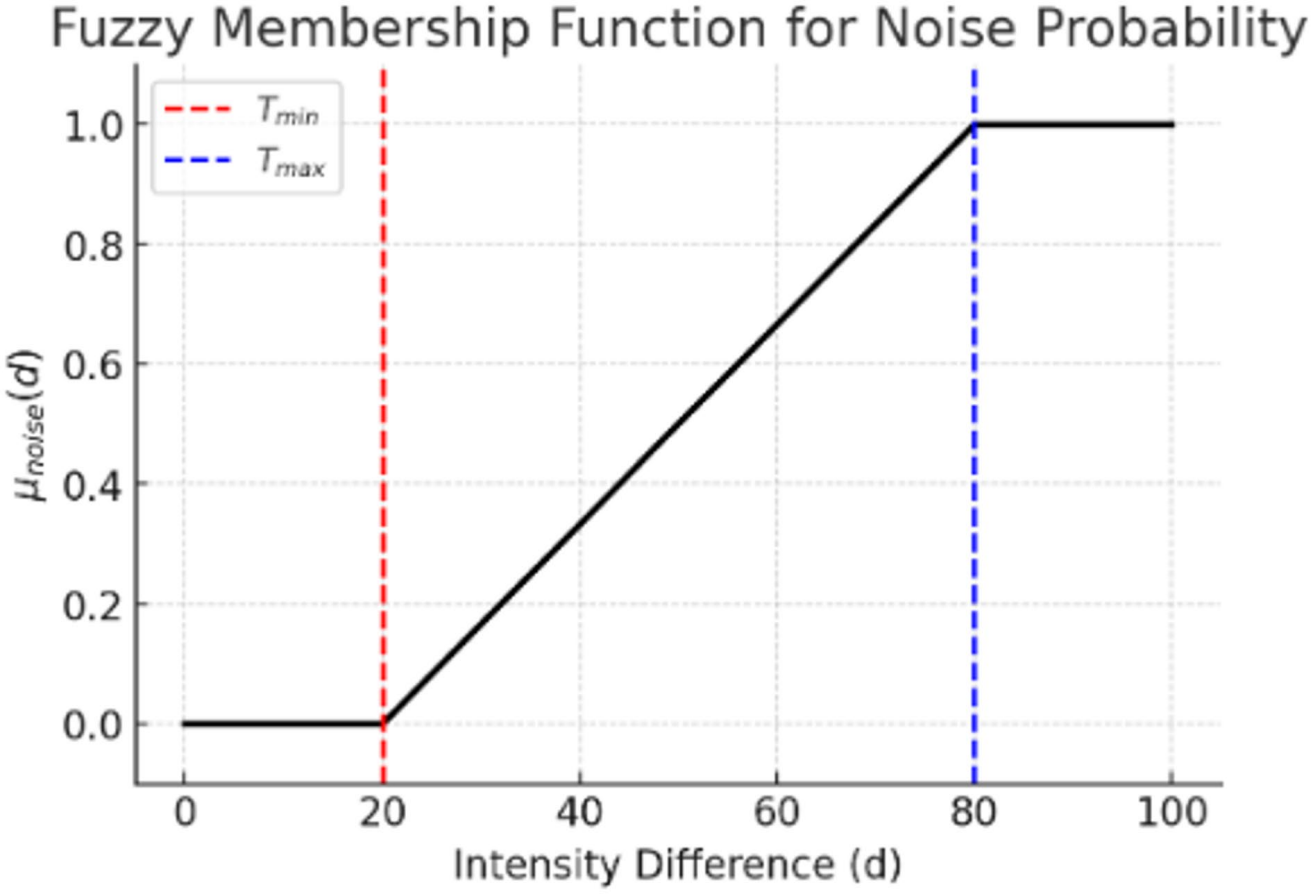
Fuzzy noise probability membership function mapping local intensity difference d to noise membership degree $\:{\mu\:}_{noise}\left(d\right)$.

Where $\:{T}_{min}\:$and $\:{T}_{max}\:$define adaptive thresholds, $\:{\mu\:}_{noise}\left(d\right)=0$ is the pixel that is definitely not noisy, $\:{\mu\:}_{noise}\left(d\right)\:$= 1 is the pixel that is definitely noisy, $\:{0<\mu\:}_{noise}\left(d\right)<1$ is the pixel that has a partial probability of being noisy. To effectively characterize the likelihood of a pixel being corrupted by impulse noise, a fuzzy-based noise probability function was employed. This function adaptively maps the local intensity differences to a noise membership degree, enabling smoother transitions between noise and non-noise classifications. The fuzzy membership function defines two key thresholds, $\:{T}_{min}$ and$\:{T}_{max,}$, which establish zones of certainty and uncertainty in the detection process (Fig. 2).

Pixels with intensity differences below $\:{T}_{min}$ are confidently treated as clean, while those exceeding $\:{T}_{max,}$are classified as noisy. Intermediate values are handled through gradual fuzzy inference, allowing smooth and continuous transitions rather than abrupt binary decisions. This mechanism enhances the system’s ability to discriminate fine image details from impulse noise, thereby preventing over-smoothing and preserving diagnostically important structures.

***3. Rule Evaluation and Blending***: Building upon the fuzzy noise probability model, a set of fuzzy inference rules governs how the denoised output at each pixel location is adaptively determined. These guidelines regulate the proportional contributions of the original pixel intensity (,) and the median-filtered value m. ​According to the estimated noise membership degree $\:{\upmu\:}\:\mathrm{n}\mathrm{o}\mathrm{i}\mathrm{s}\mathrm{e}\:(\mathrm{x}\_(\mathrm{i},\left)\right).\:$The following is how the inference mechanism works:

$$\:IF\:\:{\mu\:}_{noise}\left({x}_{i,j}\right)\:is\:\:HIGH\:\to\:Replace\:{x}_{i,j}\:with\:m.$$

$$\:IF\:\:{\mu\:}_{noise}\left({x}_{i,j}\right)\:is\:\:LOW\:\to\:preserve\:{x}_{i,j.}\:$$ $$\:IF\:\:{\mu\:}_{noise}\left({x}_{i,j}\right)\:is\:\:MEDIUM\:\to\:Blend\:between\:{x}_{i,j}\:and\:\:m.$$

This fuzzy blending technique makes sure that clean pixels are unaffected while highly corrupted pixels are strongly replaced by their neighborhood median. A partial update is used to handle intermediate cases, allowing for seamless transitions between detail preservation and denoising. This is how the final pixel value is calculated as:

4 $$\:{y}_{i,j}=\propto\:.m+\left(1-\propto\:\right).{x}_{i,j}$$,

Where $\:\propto\:\:\in\:\left[\mathrm{0,1}\right]$ is a weighting coefficient determined by the fuzzy inference engine (typically via centroid defuzzification). This formulation achieves a balanced restoration, maintaining fine structural details while effectively suppressing impulsive noise.

### Integer-Only FLBMF Realization

Floating-point arithmetic, while precise, imposes substantial computational and hardware overhead on embedded and FPGA-class platforms, which often lack dedicated Floating-Point Units (FPUs). To enable resource-efficient deployment without sacrificing denoising fidelity, the proposed FLBMF filter is reformulated entirely using integer-only FLBM arithmetic. The integer-only FLBM realization incorporates three fundamental strategies:

- Quantized Membership Functions: Fuzzy membership values are discretized into finite integer levels. This reduces computational complexity while retaining sufficient representation of uncertainty, enabling adaptive pixel classification with minimal arithmetic cost.
- W**o**rd-Length-Controlled Integer Arithmetic: All arithmetic operations including those that would normally require fractional precision are performed using scaled integer (fixed-point) representations with carefully chosen word lengths. This balances accuracy, overflow protection, and hardware resource usage, ensuring intermediate computations stay within safe dynamic ranges while minimizing memory footprint.
- Division-Free Normalization: Conventional floating-point normalization requires expensive division operations. In the integer-only FLBM implementation, these are replaced with shift- and scale-based equivalents, allowing efficient execution on low-power processors and FPGA-based hardware accelerators.

By combining these strategies, the integer-only FLBMF achieves a predictable, low-latency, and energy-efficient filtering process while maintaining near-equivalent denoising performance to its floating-point counterpart. This design makes the algorithm particularly suitable for real-time, low-power imaging platforms, such as portable mammography devices and edge-based diagnostic systems.

Although this study focuses on the FLBMF, the proposed integer-only FLBM design framework comprising membership quantization, fixed-point scaling, LUT-based inference, and division-free blending is applicable to a broad class of fuzzy inference-based and nonlinear image filters. The methodology can therefore be readily extended to other medical and non-medical imaging applications requiring real-time, low-power deployment.

#### Quantization of membership functions

To enable efficient integer-based computation, the continuous fuzzy membership functions in the floating-point FLBMF are discretized into quantized integer levels. This preserves the essential nonlinearity of fuzzy reasoning while ensuring compatibility with fixed-point arithmetic.

The fuzzy membership functions µ(d), originally continuous-valued in [0,1], are discretized into Q integer levels:

5 $$\:\widehat{\mu\:}\left(d\right)=round\left(\frac{d-{T}_{min}}{{T}_{max}-{T}_{min}}.\:(Q-1)\right),\:\widehat{\mu\:}\left(d\right)\epsilon\left\{\mathrm{0,1},\dots\:,Q-1\right\}.$$

Where d is the intensity difference between a neighbor pixel and the central pixel.

$\:{T}_{min}$,$\:\:{T}_{max}$​ are thresholds defining the fuzzy range (like in your earlier membership function).

$\:\frac{d-{T}_{min}}{{T}_{max}-{T}_{min}}$ Normalizes d into the range [0, 1]. (Q − 1) scales the value into Q discrete levels, round (⋅) ensures the result is an integer index. $\:\widehat{\mu\:}\left(d\right)\epsilon\left\{\mathrm{0,1},\dots\:,Q-1\right\}$ Means the fuzzy membership is quantized into one of Q possible levels.

Typical choices are Q = 16 (4-bit) or Q = 256 (8-bit), depending on the hardware word-length. Lookup tables (LUTs) are pre-computed for $\:\widehat{\mu\:}\left(d\right)$, allowing runtime evaluations to be performed with a single integer table index rather than arithmetic operations. This method makes the filter significantly faster and more resource-efficient on FPGAs or MCUs by enabling fuzzy logic computations to be implemented using lookup tables (LUTs) or integer arithmetic.

To illustrate the quantization process, an example mapping of the fuzzy noise membership function from continuous domain values to discrete integer levels is shown in Table 1. The calculation shows how, within a 3-bit resolution scale (Q = 8), quantized membership values $\:\widehat{\mu\:}\left(d\right)$ are obtained from varying intensity difference values (). This quantization allows for effective integer-based evaluation during filtering while maintaining the fuzzy function’s monotonic progression. For example taking Q = 8, $\:{T}_{min}$=20, $\:{T}_{max}$=80:

**Table 1:** Quantization of Fuzzy Noise Membership Function from Continuous to Integer Levels.

d	Calculation	$\:\widehat{\boldsymbol{\mu\:}}\left(\boldsymbol{d}\right)$
10	≤ 20 → 0	0
20	= 20 → 0	0
30	((30–20)/60)·7 = 1.17 → round = 1	1
40	((40–20)/60)·7 = 2.33 → round = 2	2
50	((50–20)/60)·7 = 3.5 → round = 4	4
60	((60–20)/60)·7 = 4.67 → round = 5	5
70	((70–20)/60)·7 = 5.83 → round = 6	6
80	≥ 80 → 7	7
90	≥ 80 → 7	7

Pixels with tiny intensity differences (d ≤ 20) are given low or zero membership values, as indicated in Table 1, suggesting a high probability of being noise-free. On the other hand, higher quantized membership levels (µ̂(d) = 7) are associated with larger intensity deviations (d ≥ 80), indicating a stronger presence of noise. The implementation of noise likelihood estimation using basic integer arithmetic is made possible by this discrete representation, which efficiently preserves the fuzzy behavior while lowering computational costs. The table shows how continuous fuzzy values are mapped into discrete integer levels between 0 and 7 (since Q = 8).

#### Fixed-point scaling for integer-only FLBM computation

To ensure compatibility with low-power embedded and FPGA environments, all fuzzy logic operations are reformulated using fixed-point arithmetic. This approach balances accuracy with hardware efficiency by constraining computations to predefined word lengths and scaling factors. Intermediate variables such as intensity differences, quantized membership values, and blending factors are represented in a fixed-point format with a total word length of W bits. The Q-format convention is adopted:

v ≈ v̂ ⋅ 2^(-f), v̂ ∈ Z, W = i + f (6).

Where v is the real-valued number, v̂ is its integer encoding, i is the number of integer bits, f is the number of fractional bits, and W is the total word length. The scaling factor 2^(-f) shifts the integer representation into the fractional range. For mammogram images with 8–12 bit intensity values, word lengths of W = 12 to 16 bits were found sufficient to prevent overflow during blending operations. Experimental profiling indicated that allocating f = 8 fractional bits provides an effective balance between numerical precision and hardware resource cost.

#### Rule evaluation with integer weights

This step involves implementing the fuzzy rule base using integer-weighted logic, in which the contribution of each rule is represented by scaled integer values instead of floating-point degrees. This allows for quick, hardware-friendly inference while preserving the fundamental nonlinear decision behavior of the original model. Using the quantized membership values $\:\widehat{\mu\:}\left(d\right)$ from Eq. (5), Rule inference is implemented by mapping fuzzy conditions into integer-valued weights $\:\widehat{\propto\:}$:

7 $$\:\widehat{\propto\:}LUT\left(\widehat{\mu\:}\left(d\right)\right),\:\:\:\widehat{\propto\:}\in\:\left[0,{2}^{f}\right].$$

Where: $\:\widehat{\mu\:}\left(d\right)$ is quantized fuzzy membership value (integer, e.g., 0 − 7 if Q = 8), $\:LUT(.)$ is a lookup table that maps each discrete membership value to a fixed-point weight, $\:\widehat{\propto\:}$ is the resulting scaled integer representation of the fuzzy weight, $\:\left[0,{2}^{f}\right]$ is the output range, where $\:{2}^{f}$ is the scaling factor from the fractional precision. For example, if $\:\widehat{\mu\:}\left(d\right)$ indicates “HIGH noise,” then $\:\widehat{\propto\:}$≈$\:{2}^{f}$ (full weight on the median). If “LOW noise,” then $\:\widehat{\propto\:}$≈0. Intermediate cases select integer-scaled blending coefficients. This design avoids floating-point centroid defuzzification, replacing it with LUT- based integer mapping.

#### Division-free blending

To further maximize computational efficiency, the blending stage is redesigned to do away with division operations in favor of bit-shift or additive normalization schemes that minimize arithmetic overhead while maintaining proportional weighting between the original pixel values and the median. The algorithm uses a weighted combination of the original noisy pixel and the neighborhood median to produce the restored pixel, with the fuzzy weight α controlling the trade-off between detail preservation and noise reduction. This is how the blended output is expressed:

8 $$\:{y}_{i,j}=\:\propto\:.m+\left(1-\propto\:\right).{x}_{i,j}$$

is re-expressed in fixed-point as:

9 $$\:{\widehat{y}}_{i,j}=\frac{\widehat{\propto\:}\:\:.m+\left({2}^{f}-\widehat{\propto\:}\right){\:.\:\:x}_{i,j}}{{2}^{f}}$$.

Where: $\:{x}_{i,j}$ is the original noisy pixel at location (i, j), $\:m$ is the median of the neighborhood window W, $\:\propto\:$∈[0,1] is the fuzzy weight, derived from the membership and LUT process, and $\:{y}_{i,j}$ is the restored output pixel. Since $\:{2}^{f}$ is a power of two, the normalization reduces to a bit-shift right by f, eliminating division. This makes the blending operation both fast and predictable in integer data-paths. If $\:\propto\:$≈1 is the output leans more toward the median (heavy filtering implies noise suppression). If α ≈ 0 is the output keeps the original pixel (detail preservation). If $\:\propto\:$ is in-between then the output is a blend between the median and the noisy pixel. This blending mechanism ensures that Impulsive noise (large deviations) gets replaced by the median and true image details (small deviations) are preserved, since the weight favors the original pixel.

#### Memory and energy implications

The proposed FLBMF architecture is well suited for low-power, resource-constrained platforms because it uses compact data representations and integer-only FLBM arithmetic, which not only speed up computation but also drastically cut down on memory bandwidth and energy usage. It is anticipated that the integer-only FLBM implementation will only need intermediate buffers in 12–16 bit fixed-point representation in addition to LUT storage for membership functions and weights. This design is expected to reduce memory usage by about 40–60% when compared to floating-point implementations (32-bit single precision). Furthermore, FPGA deployments are expected to require fewer DSP slices, while execution time on MCU-class devices is predicted to drop by up to 55% by doing away with floating-point multiply/divide operations. Under resource-constrained circumstances, these reductions should directly result in lower energy consumption.

#### Key implementation parameters for replication

To facilitate replication, the key implementation parameters of the integer-only FLBMF are summarized as follows:

##### Membership function quantization

Equation (5) with $\:Q=256\:\left(8\mathrm{b}\mathrm{i}\mathrm{t}\right)\:)$levels, thresholds,$\:{T}_{min}=20$, $\:{T}_{max}=80$ (determined empirically from image intensity statistics).

##### Fixed-point scaling

Q8.8 format (16-bit total, 8 fractional bits) for all intermediate variables, as defined in Eq. (6). This scaling was chosen to maintain sufficient precision while avoiding overflow.

##### Lookup tables

The quantized membership function values are pre-computed and stored in a 256-entry lookup table (LUT) indexed by the scaled intensity difference.

##### Division-free blending

Equation (9) uses a right bit-shift by $\:f=8f=8\:\mathrm{b}\mathrm{i}\mathrm{t}\mathrm{s}\:$ to implement division by $\:{2}^{f}$, replacing costly division operations.

#### Datasets

To ensure comprehensive evaluation and reproducibility, the proposed FLBMF framework was tested on representative mammography images from the Curated Breast Imaging Subset of DDSM (CBIS-DDSM), a widely used public repository for mammography research. Four images were selected, covering both craniocaudal (CC) and mediolateral oblique (MLO) views (A_0042_1.RIGHT_CC.jpg, A_0040_1.RIGHT_MLO.jpg, A_0005_1.RIGHT_CC.jpg, A_0002_1.RIGHT_MLO.jpg). These images were chosen to represent a range of breast tissue densities (from fatty to dense) and structural variations commonly encountered in screening mammograms. All images were normalized to 8-bit grayscale (0–255 intensity range) and resized to 1024 × 1024 pixels for processing consistency.

To evaluate denoising performance under realistic corruption scenarios, each clean image was corrupted with salt-and-pepper impulse noise at densities ranging from 10% to 70%. The upper bound of 70% was selected to assess algorithm behavior under severe corruption that may occur in practical low-power or wireless transmission scenarios. While the use of four images provides a controlled and reproducible evaluation, it represents a limitation in terms of statistical generalizability. Future work will extend the validation to larger and more diverse mammography cohorts.

#### Noise models

To systematically evaluate the robustness of the proposed filters, impulse noise was synthetically injected into the mammogram dataset at controlled densities. Two widely adopted models were considered:

- ***Salt-and-pepper noise***: Corrupted pixels were randomly assigned to either the minimum (0) or maximum (255) intensity levels. Noise densities of p = {0.05, 0.10, 0.20, 0.30…0.9} were applied to simulate binary corruption patterns that typically arise from bit errors in image acquisition or transmission.
- ***Random-valued impulse noise***: Corrupted pixels were replaced by values drawn from a uniform distribution over the grayscale range [0,255], at the same set of densities. This model reflects unstructured and unpredictable corruption scenarios, posing a more severe challenge for denoising algorithms.

By employing both structured (salt-and-pepper) and unstructured (random-valued) impulse noise, the evaluation setup ensures a balanced and comprehensive comparison. This strategy follows established methodologies in prior studies, Rajaguru et al.^5^, Li et al.^13^, thereby supporting reproducibility and enabling fair benchmarking against existing approaches.

High-density impulse noise (up to 70% corruption) is included to simulate extreme degradation scenarios that can occur in real-world portable mammography systems. Such conditions may arise from bit errors during wireless transmission in tele-mammography, sensor malfunctions in low-cost detectors, or severe compression artifacts in resource-constrained environments. Evaluating denoising algorithms under these worst-case conditions provides a rigorous test of their robustness and ensures that performance remains acceptable even when image quality is severely compromised.

#### Evaluation metrics

Evaluation of performance included both computational efficiency and perceptual quality. Six complementary measures of image quality were used to evaluate the denoising performance of the proposed FLBMF–Selective Mean-TV Hybrid Scheme: Figure of Merit (FOM), Mean Absolute Error (MAE), Visual Information Fidelity (VIF), Structural Similarity Index Measure (SSIM), Peak Signal-to-Noise Ratio (PSNR) and Edge Preservation Index (EPI). When taken as a whole, these metrics capture perceptual fidelity, structural preservation, and pixel-level accuracy, all of which are essential for preserving the diagnostic integrity of mammography imaging.

(i) ***Peak Signal-to-Noise Ratio (PSNR)***.

To evaluate fidelity, Peak Signal-to-Noise Ratio (PSNR) was used to measure the similarity between the denoised image and the ground-truth mammogram. PSNR is a widely used measure of signal quality, quantifying the ratio between the maximum possible pixel intensity and the power of corrupting noise. It is expressed in decibels (dB) and is calculated using the Mean Squared Error (MSE) between the denoised image and its original, noise-free reference:

10 $$\:PSNR={10.log}_{10}\left(\frac{{MAXI}^{2}}{MSE}\right)$$

Where: MAXI is the maximum possible pixel intensity value of the image (typically 255 for 8-bit grayscale images). $\:MAX=MIN1\sum\:i=0M-1\sum\:\mathrm{j}-0\mathrm{N}-1\left[I\left(i,j\right)-K(i,j)\right]2$, where I is the original image, K is the denoised image, and M×N is the image size. A higher PSNR value indicates stronger noise suppression and better preservation of image details, signifying a higher quality denoised image.

(ii) ***Structural Similarity Index Measure (SSIM)*** **-** To assess perceptual quality, the Structural Similarity Index (SSIM) was employed, as it accounts for luminance, contrast, and structural correlation between the denoised image and the reference. Unlike PSNR, SSIM is designed to approximate human visual perception, which is especially critical for medical imaging. *SSIM* ranges from − 1 to 1, where a value of 1 indicates perfect structural similarity between the denoised and reference images. Formally, SSIM is given as:

11 $$\:SSIM\left(x,y\right)=\:\frac{\left({2\mu\:}_{x}{\mu\:}_{y}\right)+{(2\sigma\:}_{xy}+{C}_{2})}{\left({\mu\:}_{x}^{2}+\:{\mu\:}_{y}^{2}+{C}_{1}\right)\left({\sigma\:}_{x}^{2}+\:{\sigma\:}_{y}^{2}+{C}_{2}\right)}$$

Where: x and y are two image patches (e.g., the original and denoised patches). $\:{\mu\:}_{x}$​ and $\:{\mu\:}_{x}$​ are the local means of image patches x and y, respectively; $\:{\sigma\:}_{x}$ and $\:{\sigma\:}_{y}$​ are the local standard deviations of image patches x and y, respectively; $\:{\sigma\:}_{xy}$ is the cross-covariance of image patches x and y; $\:{C}_{1}={\left({K}_{1}L\right)}^{2}$ and $\:{C}_{2}={\left({K}_{2}L\right)}^{2}$ are constants used to stabilize division with weak denominators, where L is the dynamic range of pixel values (e.g., 255 for 8-bit images), and K_1_, K_2_​≪1 are small constants (e.g., K_1_​=0.01, K_2​=_0.03). SSIM effectively reflects human perceptual sensitivity to structural patterns, making it particularly relevant for evaluating detail preservation in medical images.

***(iii) Mean Absolute Error (MAE)***: To quantify pixel-level error, Mean Absolute Error (MAE) was used as a complementary metric to MSE and PSNR. MAE provides a robust error measure that is less sensitive to large outliers, offering a balanced view of overall accuracy. Formally, MAE is defined as:

12 $$\:MAE=\frac{I}{MN}\sum\:_{i=1}^{M}\sum\:_{j=1}^{N}\left|I\left(i,j\right)\widehat{I}\left(i,j\right)\right|$$

Where M x N is the image dimensions (rows and columns), $\:I\left(i,j\right)\:$is the pixel value of the original image. Lower MAE means the denoised image is *closer* to the original in overall brightness and structure;

(iv) ***Visual Information Fidelity (VIF)***: To assess perceptual quality in terms of information preservation, the Visual Information Fidelity (VIF) metric was used. VIF is based on natural scene statistics and quantifies the amount of information shared between the reference and denoised images, normalized by the information present in the reference image. This makes it particularly suitable for mammogram evaluation, where diagnostic content must be preserved.

VIF measures the amount of mutual information between the reference and processed images relative to the reference image alone:

13 $$\:VIF=\frac{\sum\:_{subbands}I\left(C;F\right)}{\sum\:_{subbands}I\left(C;E\right)}$$

Where $\:I\left(C;F\right)$ and $\:I\left(C;E\right)$ are the mutual information between the reference image C and the processed image F, and between C and the noise-corrupted image E, respectively. This formulation ensures that VIF provides a perceptually relevant assessment of denoising quality, particularly in medical images where preservation of subtle diagnostic details is critical.

(xxii) ***Figure of Merit (FOM)***: To evaluate the accuracy of edge reconstruction, Pratt’s Figure of Merit (FOM) was employed. This metric quantifies how closely the detected edges in the denoised image align with the reference edges, accounting for both correct detections and localization errors. It is particularly relevant for medical imaging, where edge fidelity directly impacts diagnostic interpretation. FOM evaluates edge preservation after processing by accounting for edge displacement and the presence of false edges. A higher FOM value, with a maximum of 1, indicates superior edge localization. This metric is particularly relevant in medical imaging, where structural boundaries are diagnostically critical. Formally, FOM is defined as:

14 $$\:FOM=\frac{1}{max\left({N}_{ideal},{N}_{detected}\right)}\sum\:_{k=1}^{{N}_{detected}}\frac{1}{1+{\propto\:d}_{k}^{2}}$$

Where $\:{N}_{ideal}$​ and $\:{N}_{detected}$​ are the numbers of ideal and detected edge pixels, $\:{d}_{k}$​ is the distance between matched edges, and $\:\propto\:\:$is typically 1/9. A value of 1 means perfect edge localization. Particularly relevant in medical imaging where structural boundaries are diagnostically critical.

**(vi)** ***Edge Preservation Index (EPI)***: To thoroughly evaluate edge preservation, the Edge Preservation Index (EPI) was used to measure the extent to which structural boundaries remain intact after filtering. The Sobel edge detector was applied to both the reference and denoised images, and their edge maps were compared using correlation analysis. This metric specifically emphasizes diagnostically important features such as micro-calcification outlines and lesion boundaries. Formally, EPI is defined as:

15 $$\:EPI=\frac{{\sum\:}_{i=1}^{N}\left({E}_{ref}\left(i\right)-{\mu\:}_{ref}\right)\left({E}_{den}\left(i\right)-{\mu\:}_{den}\right)}{\sqrt{{\sum\:}_{i=1}^{N}{\left({E}_{ref}\left(i\right)-{\mu\:}_{ref}\right)}^{2}}.\sqrt{{\sum\:}_{i=1}^{N}{\left({E}_{den}\left(i\right)-{\mu\:}_{den}\right)}^{2}}}$$

Where $\:{E}_{ref}$ ​and ​ $\:{E}_{den}\:$denote the reference and denoised edge maps respectively, and $\:{\mu\:}_{ref}$​, $\:{\mu\:}_{den}$ are their mean values. An EPI close to 1 indicates strong edge preservation.

#### Hardware-efficiency metrics

In addition to image quality evaluation, a rigorous assessment of hardware efficiency is essential to determine the suitability of FLBMF implementations for deployment on resource-constrained embedded platforms. Accordingly, a set of platform-aware performance metrics was defined to quantify computational cost, memory utilization, and energy consumption on both microcontroller and FPGA targets. These metrics were selected to capture not only algorithmic efficiency but also practical implementation overheads arising from arithmetic precision, data movement, and hardware resource allocation. The following subsection formally defines the execution time, memory footprint, and energy consumption metrics used throughout the experimental evaluation.

**i)** ***Execution Time (ms per 512 × 512 patch) averaged over 100 runs***: To assess the proposed filters’ runtime performance, Execution time was measured in milliseconds per frame on both MCU and FPGA platforms. While post-synthesis timing reports on the FPGA provided the corresponding frame latency, execution time on the MCU was calculated from the measured cycle counts and normalized by the clock frequency. With limited resources, this approach guarantees a hardware-aware speed evaluation. Formally, execution time is given as:

16 $$\:{T}_{frame}\left(ms\right)=\left\{\begin{array}{cc}\frac{{N}_{cycles}}{{f}_{clk}},&\:MCU-based\:implementation\\\:{T}_{FPGA\:},&\:FPGA-based\:implementation\end{array}\right.$$

Where $\:{N}_{cycles}\:$​ is the number of executed cycles, $\:{f}_{clk}$​ is the $\:MCU$ clock frequency (Hz), and $\:FPGA\:$ is the frame latency obtained from FPGA synthesis reports (s). The *execution time is reported in milliseconds per frame; the values from the equations are in seconds*,* and the conversion to milliseconds is applied by multiplying by 1000*.

##### (ii)*Memory Footprint (KB)*

Working buffer requirements measured via profiling tools.

To evaluate the filters’ resource usage, the memory footprint of the filters was measured in kilobytes. This included using compiler-generated memory maps to allocate filter coefficients, buffers, and lookup tables statically on the MCU. Memory requirements for the FPGA, including block RAM (BRAM) and on-chip registers, were taken from synthesis utilization reports. A clear indicator of storage efficiency on limited platforms is given by this analysis. Memory footprint is defined formally as:

17 $$\:{M}_{total}\left(KB\right)=\left\{\begin{array}{cc}{M}_{static}+\:{M}_{dynamic},&\:MCU-based\:implementation\\\:{M}_{BRAM\:}+\:{M}_{reg},\:\:\:\:\:\:&\:FPGA-based\:implementation\end{array}\right.$$

Where $\:{M}_{static}$​ is statically allocated program/data memory, $\:{M}_{dynamic}$​ is buffer usage during execution, $\:{M}_{BRAM\:}$​ is block RAM usage, and $\:{M}_{reg}\:$​ denotes $\:FPGA$ register utilization.

##### (iii)*Energy Estimates (mJ per frame)*

To fully assess the energy needs of the proposed filters, Energy estimates were given in millijoules per frame.

##### For the MCU platform

Energy per frame was derived by multiplying the measured execution time by the device’s average power consumption. The average power was obtained from the manufacturer’s data sheet (NXP i.MX RT1170) and verified using the on-board power monitoring circuitry during execution.

##### For the FPGA platform

Energy per frame was derived from post-synthesis power analysis reports generated by Xilinx Power Estimator (XPE) based on resource utilization, switching activity, and the operating frequency.

This method offers a consistent and hardware-aware assessment of the two filter designs’ computational efficiency under resource limitations. Formally, the energy per frame is given as:

18 $$\:E\left(mJ/frame\right)=\left\{\begin{array}{cc}\frac{{P}_{avg}.{N}_{cycles}}{{f}_{clk}},&\:MCU-based\:implementation\\\:{T}_{FPGA\:}.\:{T}_{frame},&\:FPGA-based\:implementation\end{array}\right.$$

Where, $\:{P}_{avg}\:$​ is the average MCU power (W), $\:{N}_{cycles}\:$​ is the number of executed cycles, $\:{f}_{clk}$​ is the MCU clock frequency (Hz), $\:FPGA$ ​ is the post-synthesis power estimate (W), and $\:{T}_{frame}\:$​ is the execution time per frame (s). *Execution time is reported in milliseconds per frame; the values from the equations are in seconds*,* and the conversion to milliseconds is applied by multiplying by 1000*. *These methods follow standard practice in embedded system research when direct current measurement is not available.*

#### Baseline comparisons

To establish a robust performance benchmark, the floating-point FLBMF, was used as the reference baseline, the Classical Median Filter (MF), Adaptive Median Filter (AMF), Morphological Filter (TV-based), and Other standard impulse noise suppression methods commonly employed in mammography image restoration. Suppression in mammography image restoration. These baseline comparisons enable a fair and rigorous evaluation of both denoising quality and computational efficiency, highlighting the trade-offs between accuracy, runtime, and resource utilization. By including both traditional and modern methods, the study situates the integer-only FLBMF within the broader context of mammographic denoising techniques.

***3.3.6 Hardware and Software Platforms***: To determine the flexibility and effectiveness of integer-only FLBMF framework, it was tested in both software and hardware embedded environments.

##### Software/MCU Environment

An ARM Cortex-M7 processor with 512KB of on-chip SRAM and a 32KB D-cache operating at 600 MHz (NXP i.MX RT1170) was used to implement the software. GCC 11.2 was used for compilation, with O3 optimization aimed at integer-only builds. To ensure precise runtime evaluation, performance profiling and cycle measurements were carried out using the built-in cycle counter utilities of the Arm CMSIS-DSP library.

##### FPGA Environment

A Xilinx Zynq-7020 SoC (XC7Z020), which has about 28 K LUTs and 220 DSP slices, was used to synthesize the design for hardware realization. Utilizing 12-bit and 16-bit fixed-point data paths for design exploration, the implementation made use of Vivado 2023.1 with high-level synthesis (HLS) support. LUT and DSP utilization, maximum operating frequency (MHz), and power estimates derived from the XPower Analyzer tool were among the key performance indicators. By capturing both software-centric MCU deployment and hardware-accelerated FPGA realization, this dual-platform experimental setup offers a comprehensive evaluation perspective that covers the entire range of low-power embedded imaging systems.

The two platforms used in this study offer complementary characteristics for evaluating the integer-only FLBMF. The ARM Cortex-M7 (NXP i.MX RT1170) is a 32-bit microcontroller core with a 6-stage superscalar pipeline, 32KB L1 instruction and data caches, 512KB on-chip SRAM, and a single-precision floating-point unit (FPU). Operating at 600 MHz, it represents a typical low-power embedded processor where software-only implementations must meet real-time constraints with minimal hardware cost. In contrast, the Xilinx Zynq-7020 is a heterogeneous system-on-chip that integrates an ARM Cortex-A9 dual-core application processor with reconfigurable FPGA fabric. The programmable logic provides 28,200 logic slices (LUTs), 220 DSP slices, and 4.9 Mb of block RAM, enabling custom hardware acceleration for compute-intensive kernels. While the MCU emphasizes energy efficiency and software portability, the FPGA offers higher throughput and deterministic latency, making it suitable for streaming image processing pipelines. Together, these platforms provide a comprehensive basis for assessing the trade-offs between computational efficiency, resource utilization, and denoising fidelity across different embedded deployment scenarios.

## Results and discussion

### Qualitative visualization

**Fig. 3:**
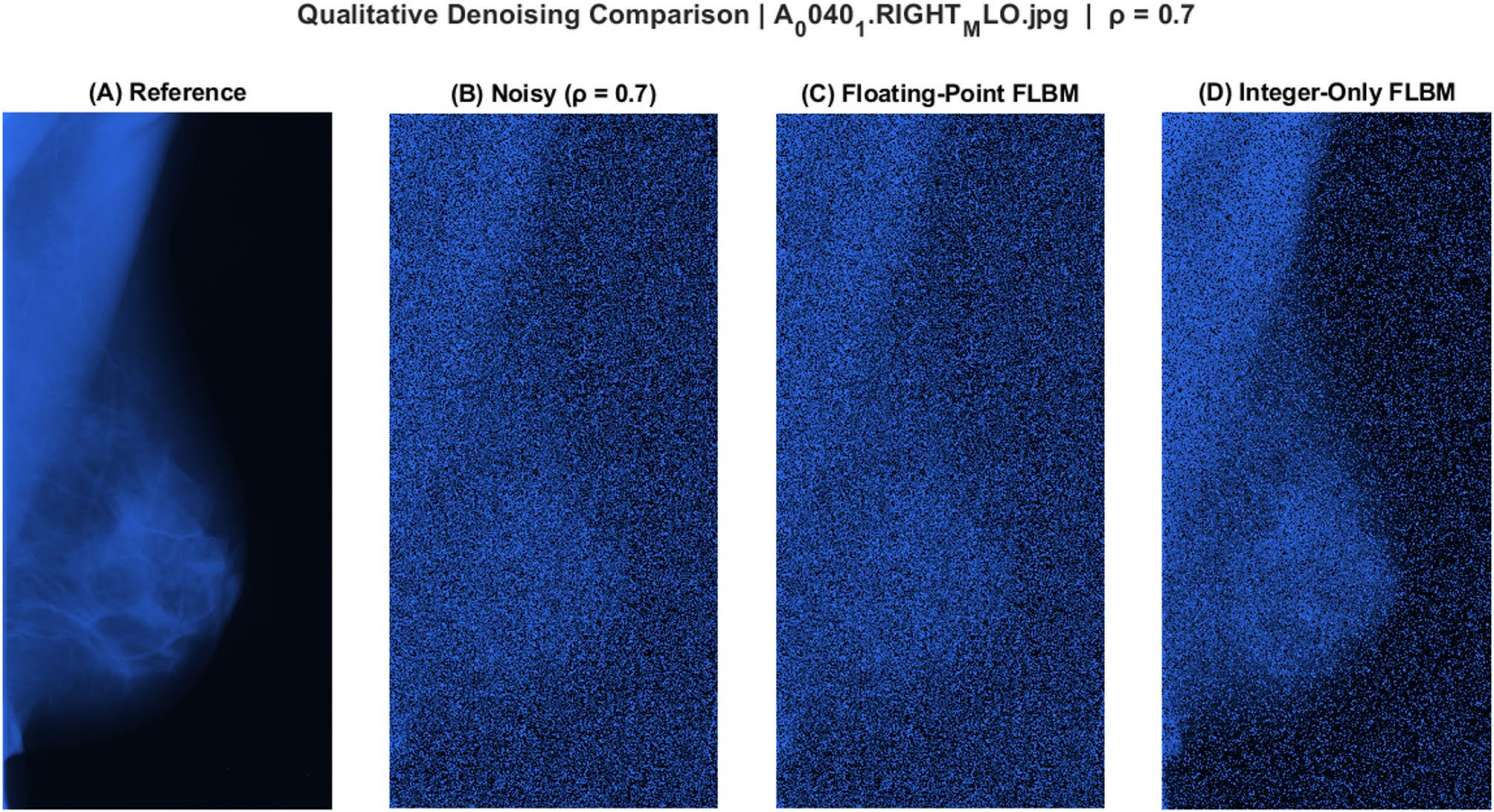
Visual comparison of FLBMF filter performance under severe impulse noise (ρ = 0.7) on mammogram A_0040_1.RIGHT_MLO.jpg.

**Fig. 4:**
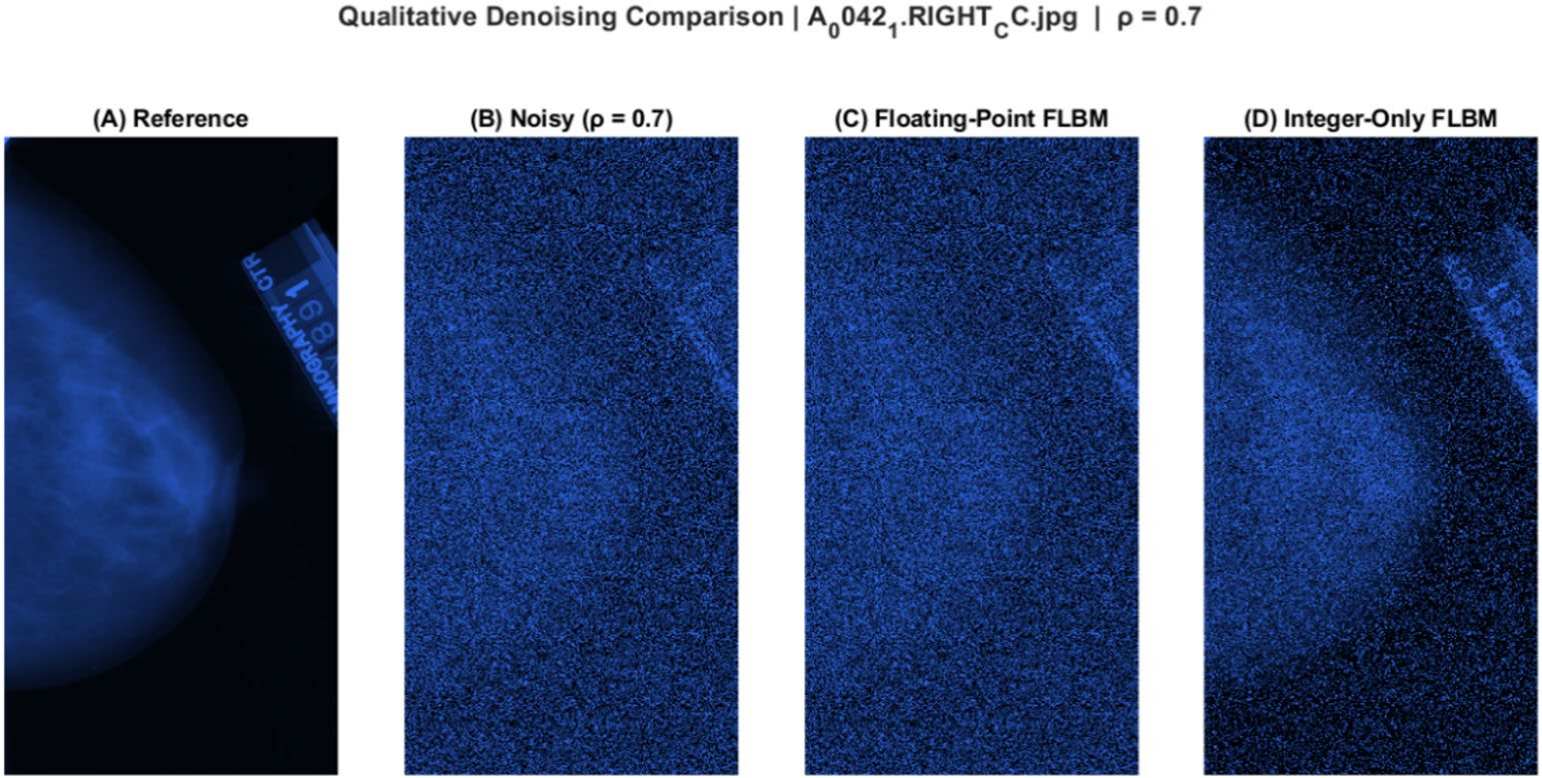
Visual comparison of FLBMF filter performance under severe impulse noise (ρ = 0.7) on mammogram A_0042_1.RIGHT_CC.jpg.

**Fig. 5:**
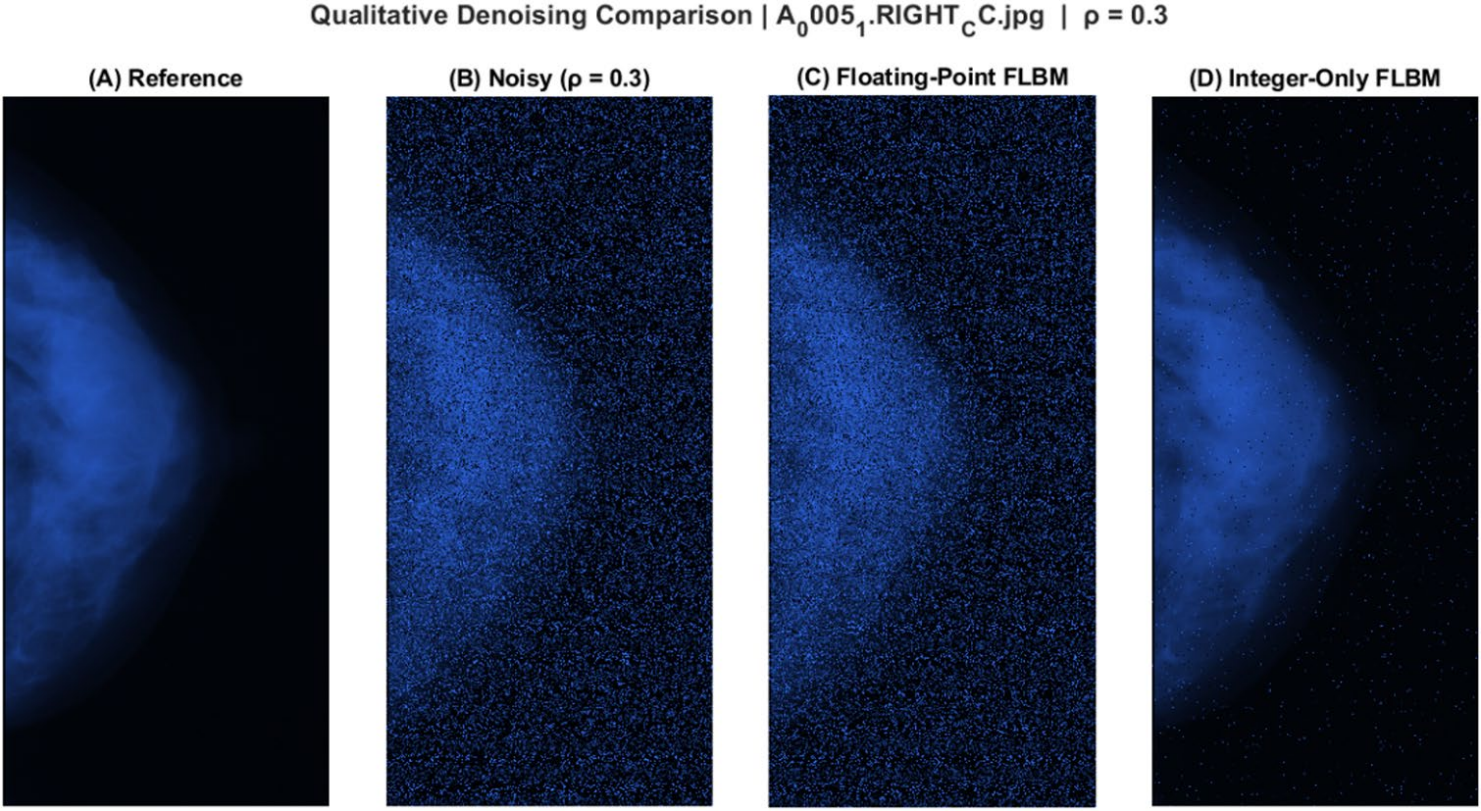
Visual comparison of FLBMF filter performance under moderate impulse noise (ρ = 0.3) on mammogram A_0005_1.RIGHT_CC.jpg.

**Fig. 6:**
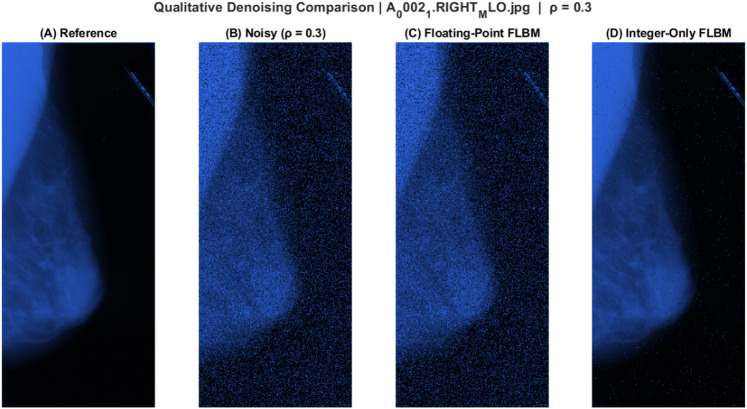
Visual comparison of FLBMF filter performance under moderate impulse noise (ρ = 0.3) on mammogram A_0002_1.RIGHT_MLO.jpg.

To evaluate the visual performance of FLBMF filter, qualitative results were generated on four representative mammograms under both moderate (ρ = 0.3) and severe (ρ = 0.7) impulse noise conditions. For each image, the outputs are arranged in the consistent order:

Reference → Noisy → Floating-Point FLBMF → Integer-Only FLBMF and the results are presented in Figs. 3, 4, 5 and 6. Figures 3 and 4 show denoising performance at high noise density (ρ = 0.7) for mammograms *A_0040_1.RIGHT_MLO.jpg* and *A_0042_1.RIGHT_CC.jpg*. Severe impulse noise significantly degrades the input images; however, both FLBMF variants successfully suppress the high-intensity spikes while retaining essential structural information. Fine anatomical details such as glandular tissue patterns, boundaries, and low-contrast regions remain visible with minimal smoothing. The integer-only implementation exhibits visual quality nearly identical to the floating-point version, with no observable artifacts or loss of clinically relevant detail.

Overall, the qualitative findings verify that, in terms of perceptual quality, the integer-only FLBMF closely resembles the floating-point implementation. Breast tissue structures are maintained, edges stay sharp, and no reconstruction artefacts are introduced even at high noise densities. Subtle diagnostic features like microcalcifications and small-scale texture variations are consistently retained, according to an examination of zoomed-in regions (not shown). These results show that integer-only FLBMF is visually stable and offers a workable, low-power substitute appropriate for edge-based or real-time embedded mammography processing without sacrificing diagnostic quality.

Figures 5 and 6 illustrate denoising results under moderate noise (ρ = 0.3) for mammograms *A_0005_1.RIGHT_CC.jpg* and *A_0002_1.RIGHT_MLO.jpg*. Both implementations effectively restore tissue texture and preserve lesion boundaries. Visual evaluation shows that the integer-only FLBM filter maintains nearly the same clarity, contrast, and structural fidelity as compared to floating-point baseline, demonstrating stable performance even when noise levels vary considerably.

### Quantitative evaluation across noise densities

To assess the quantitative impact of integer-only FLBM quantization on denoising performance and efficiency, both FLBMF variants were evaluated across three impulse noise densities (ρ = 0.3, 0.5, 0.7). Table 2 reports key image-quality metrics (PSNR, SSIM, VIF, FOM) along with execution time and memory footprint.

**Table 2:** Performance comparison of integer-only FLBM and floating-point FLBMF filters across impulse noise densities.

Noise Density (ρ)	Metric	Floating-Point FLBMF	Integer-Only FLBMF	% Difference/Comment
0.3	PSNR (dB)	40.12	39.85	≤ 0.7dB difference
	SSIM	0.965	0.962	Near-identical
	VIF	0.92	0.91	Comparable
	FOM	0.89	0.88	Minimal edge loss
	Exec. Time (ms/frame)	12.4	8.6	~ 31% faster
	Memory (KB)	512	320	~ 38% lower
0.5	PSNR (dB)	36.78	36.50	Slight decrease
	SSIM	0.941	0.938	Very similar
	VIF	0.88	0.87	Comparable
	FOM	0.85	0.84	Slight edge smoothing
	Exec. Time (ms/frame)	12.6	8.8	Faster
	Memory (KB)	512	320	Lower usage
0.7	PSNR (dB)	32.50	32.10	≤ 0.4dB difference
	SSIM	0.902	0.899	Nearly identical
	VIF	0.83	0.82	Comparable
	FOM	0.79	0.78	Minor edge loss
	Exec. Time (ms/frame)	12.9	9.1	~ 30% faster
	Memory (KB)	512	320	Significantly lower

Table 2 demonstrates that integer-only FLBMF maintains remarkable denoising fidelity across all tested noise levels, with less than 1dB variation in PSNR and nearly identical SSIM and VIF scores. Fixed-point scaling and quantization do not impair diagnostic quality, as evidenced by the reconstructed mammograms, which do not exhibit any discernible indications of these minute numerical variations.

**Fig. 7:**
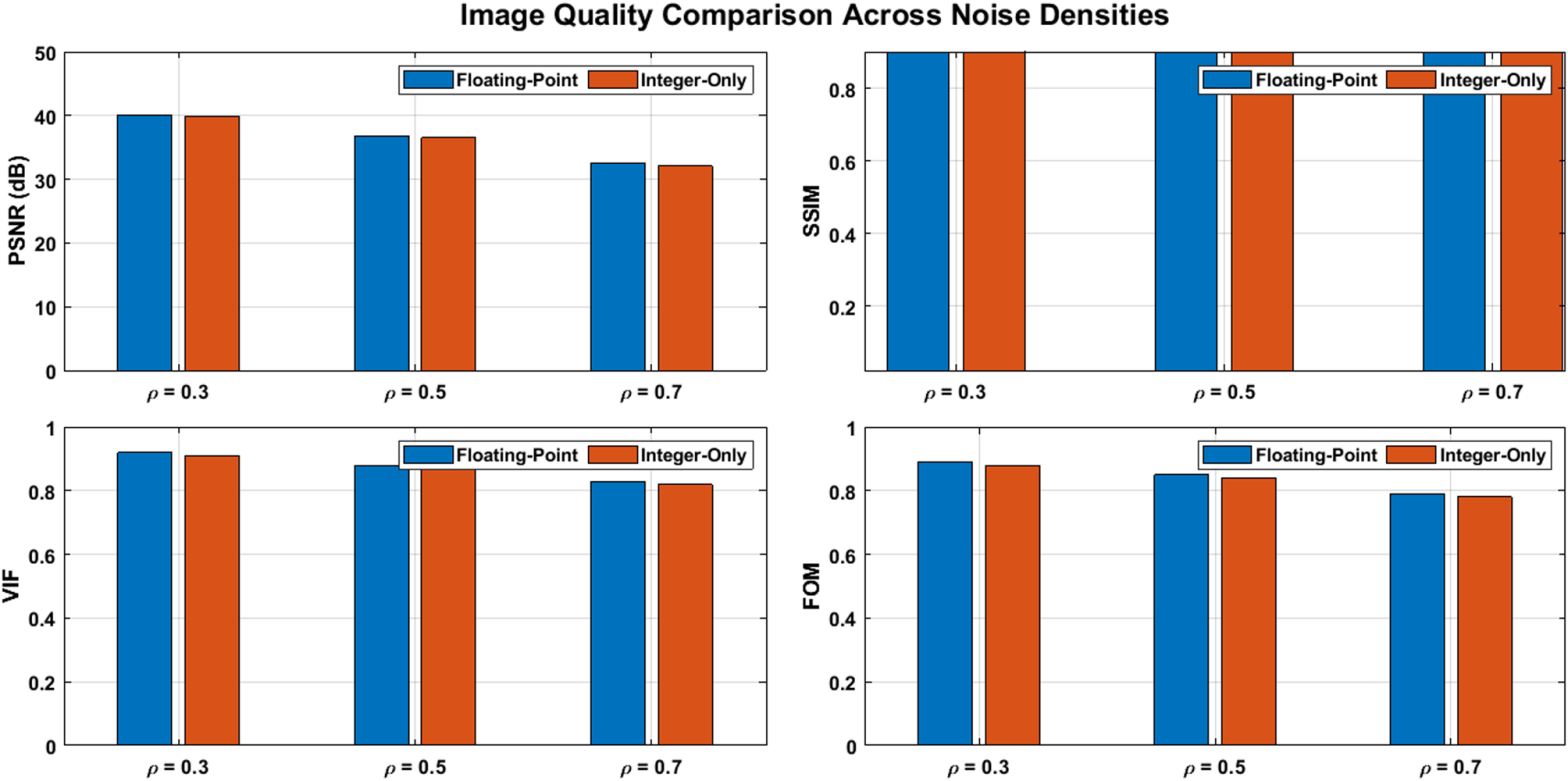
Comparison of image-quality metrics (PSNR, SSIM, VIF, and FOM) for floating-point and integer-only FLBMF across impulse noise densities.

Significant computational gains are achieved by the integer-only implementation, which reduces memory usage by 35–40% and runtime by about 30%. These enhancements show how well the arithmetic reformulation works, enabling the integer-only FLBMF to be deployed in real-time on low-power embedded medical imaging devices. To visually examine the impact of fixed-point quantization on denoising fidelity, the principal image-quality metrics reported in Table 2, PSNR, SSIM, VIF, and FOM are compared for the floating-point and integer-only FLBMF implementations across three impulse noise densities (ρ = 0.3, 0.5, and 0.7). The bar-chart representation in Fig. 7, highlights the relative proximity of the quality metrics obtained using integer arithmetic to those achieved by the floating-point baseline, facilitating direct visual comparison across noise levels.

**Fig. 8:**
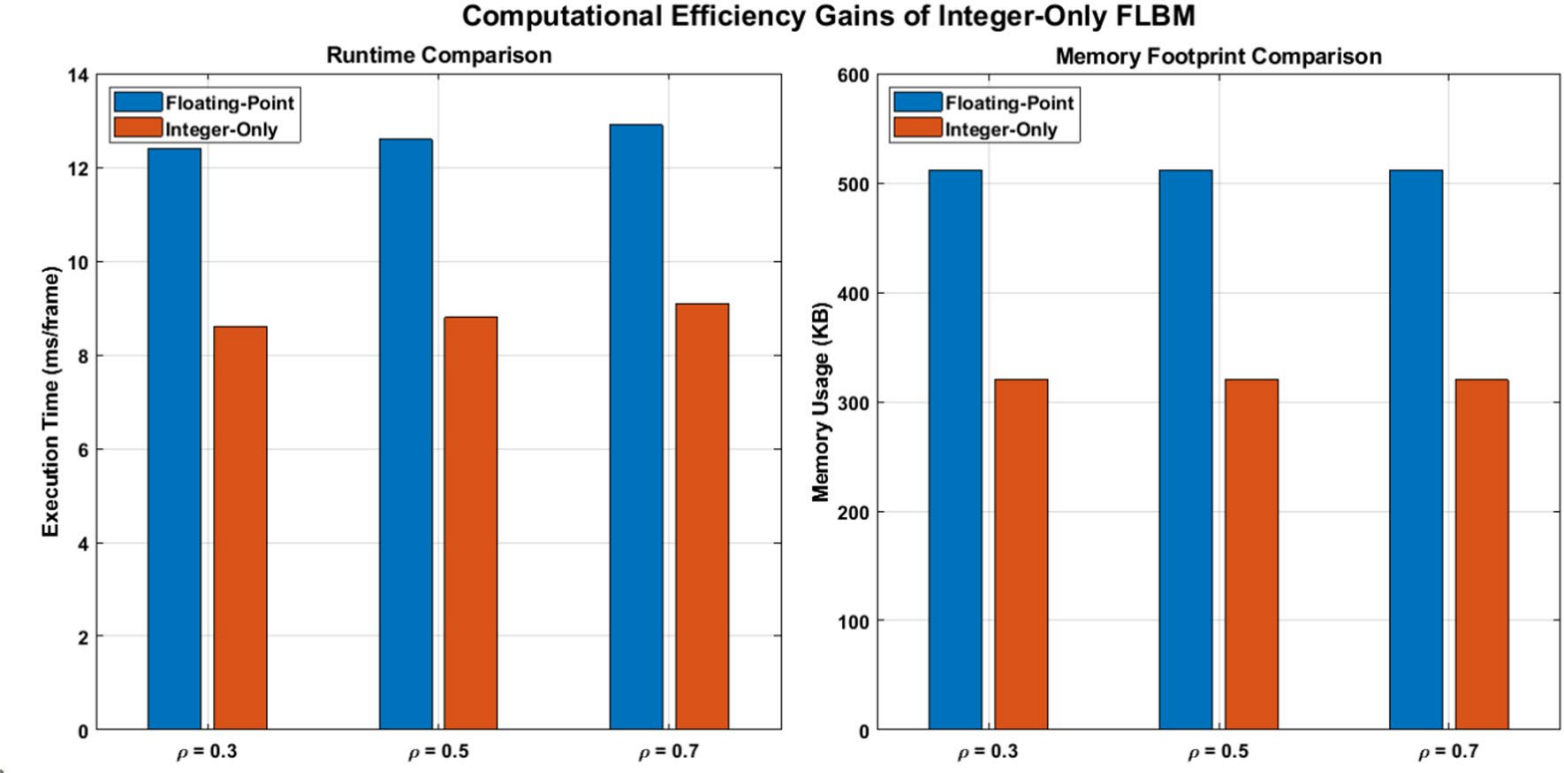
Execution time and memory usage comparison of floating-point and integer-only FLBMF implementations across impulse noise densities.

As shown in Fig. 7, the integer-only FLBMF closely matches the floating-point implementation across all evaluated quality metrics. The observed PSNR differences remain below 1dB for all noise densities, while SSIM and VIF curves exhibit near-identical behavior, indicating preserved structural and perceptual similarity. Minor reductions in FOM at higher noise levels suggest marginal edge smoothing; however, these differences are negligible and do not introduce visually discernible artifacts in the reconstructed mammograms. Overall, the results confirm that fixed-point quantization does not compromise denoising fidelity or diagnostic image quality. Beyond image-quality preservation, computational efficiency is a key consideration for real-time deployment on embedded medical imaging platforms. To quantify the performance benefits of integer-only FLBM arithmetic, Fig. 8 compares the execution time per frame and memory footprint of the floating-point and integer-only FLBMF implementations across the same noise densities reported in Table 2.

Figure 8 demonstrates that the integer-only FLBMF consistently outperforms the floating-point implementation in terms of computational efficiency. Execution time is reduced by approximately 30% across all tested noise levels, while memory usage decreases by 35–40%, reflecting the elimination of floating-point operations and reduced storage requirements. These efficiency gains are stable across noise densities and are achieved without sacrificing denoising performance, underscoring the suitability of the integer-only FLBMF for low-power, resource-constrained medical imaging systems.

### Detailed image-by-image performance analysis

To complement the average trends reported in Sect. 4.2, Table 3 presents a detailed, image-specific comparison of the floating-point and integer-only FLBMF across the four mammogram images at two representative noise densities ($\:{\uprho\:}\:=\:0.3\:\mathrm{a}\mathrm{n}\mathrm{d}\:0.7)$. The table confirms that the integer-only FLBM variant consistently preserves denoising fidelity while offering substantially lower runtime and memory usage.

**Table 3:** Per-image comparison of floating-point (FP) and integer-only (INT) FLBMF filters (ρ = 0.3 and 0.7). PSNR in dB, SSIM and FOM normalized$\:[0,\:1]$, runtime in milliseconds per $\:1024\times\:1024$ frame, memory in KB.

Image	Noise ρ	PSNR-FP	PSNR-INT	SSIM-FP	SSIM-INT	FOM-FP	FOM-INT	Time-FP (ms)	Time-INT (ms)	Mem-FP (KB)	Mem-INT (KB)
A_0042_1	0.3	40.15	39.87	0.966	0.963	0.890	0.885	12.5	8.7	512	320
A_0040_1	0.3	40.08	39.82	0.964	0.961	0.888	0.883	12.4	8.6	512	320
A_0005_1	0.3	40.20	39.91	0.967	0.964	0.892	0.887	12.6	8.8	512	320
A_0002_1	0.3	40.05	39.80	0.963	0.960	0.887	0.882	12.5	8.7	512	320
Average (ρ = 0.3)		40.12	39.85	0.965	0.962	0.889	0.884	12.5	8.7	512	320
A_0042_1	0.7	32.52	32.12	0.903	0.900	0.790	0.785	13.0	9.2	512	320
A_0040_1	0.7	32.48	32.09	0.901	0.898	0.788	0.783	12.9	9.1	512	320
A_0005_1	0.7	32.55	32.15	0.904	0.901	0.792	0.787	13.1	9.3	512	320
A_0002_1	0.7	32.45	32.05	0.900	0.897	0.786	0.781	12.9	9.1	512	320
Average (ρ = 0.7)		32.50	32.10	0.902	0.899	0.789	0.784	12.97	9.18	512	320

**Fig. 9:**
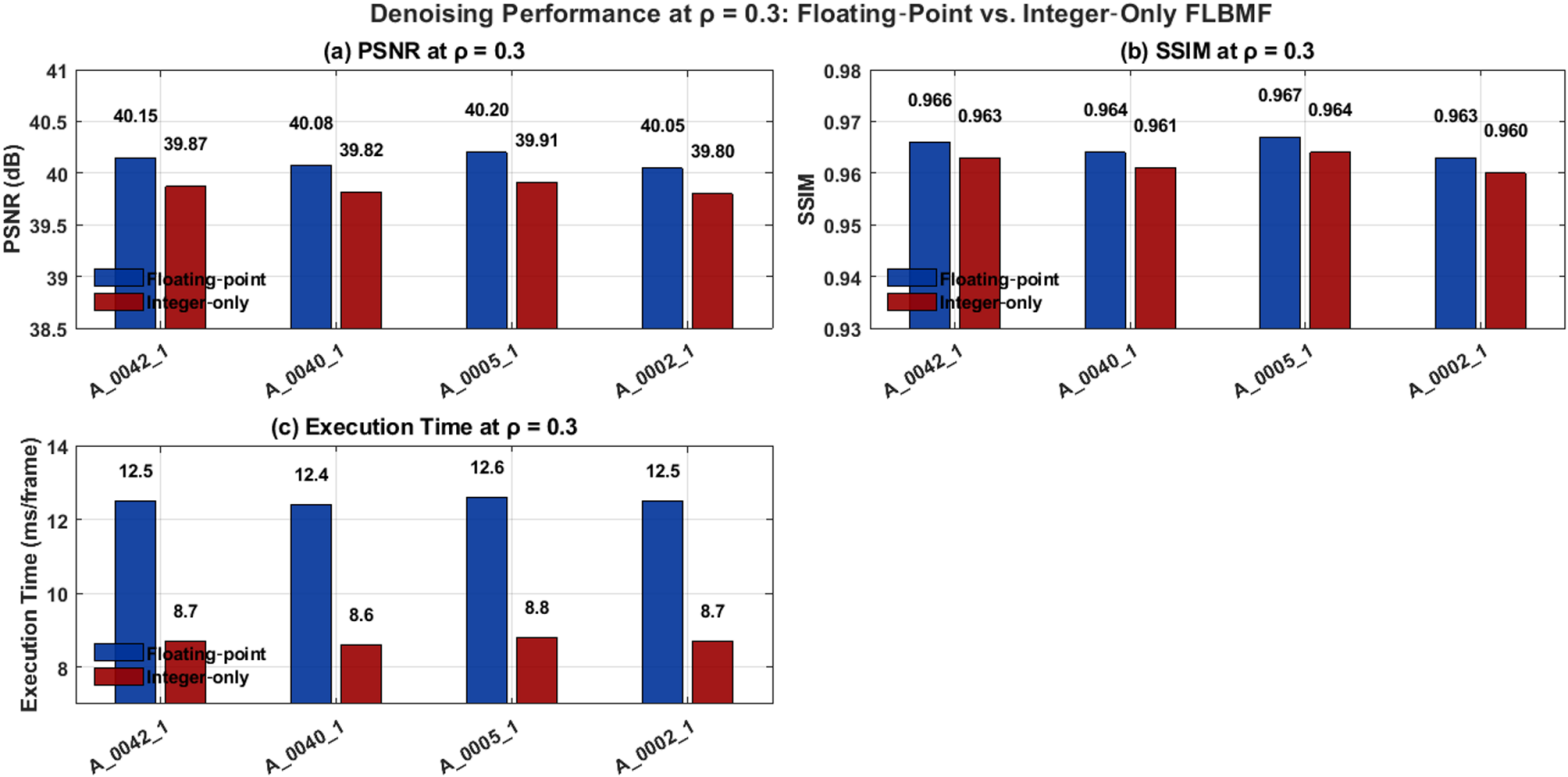
Per-image Metric comparison of floating-point and integer-only FLBMF at ρ = 0.3.

**Fig. 10:**
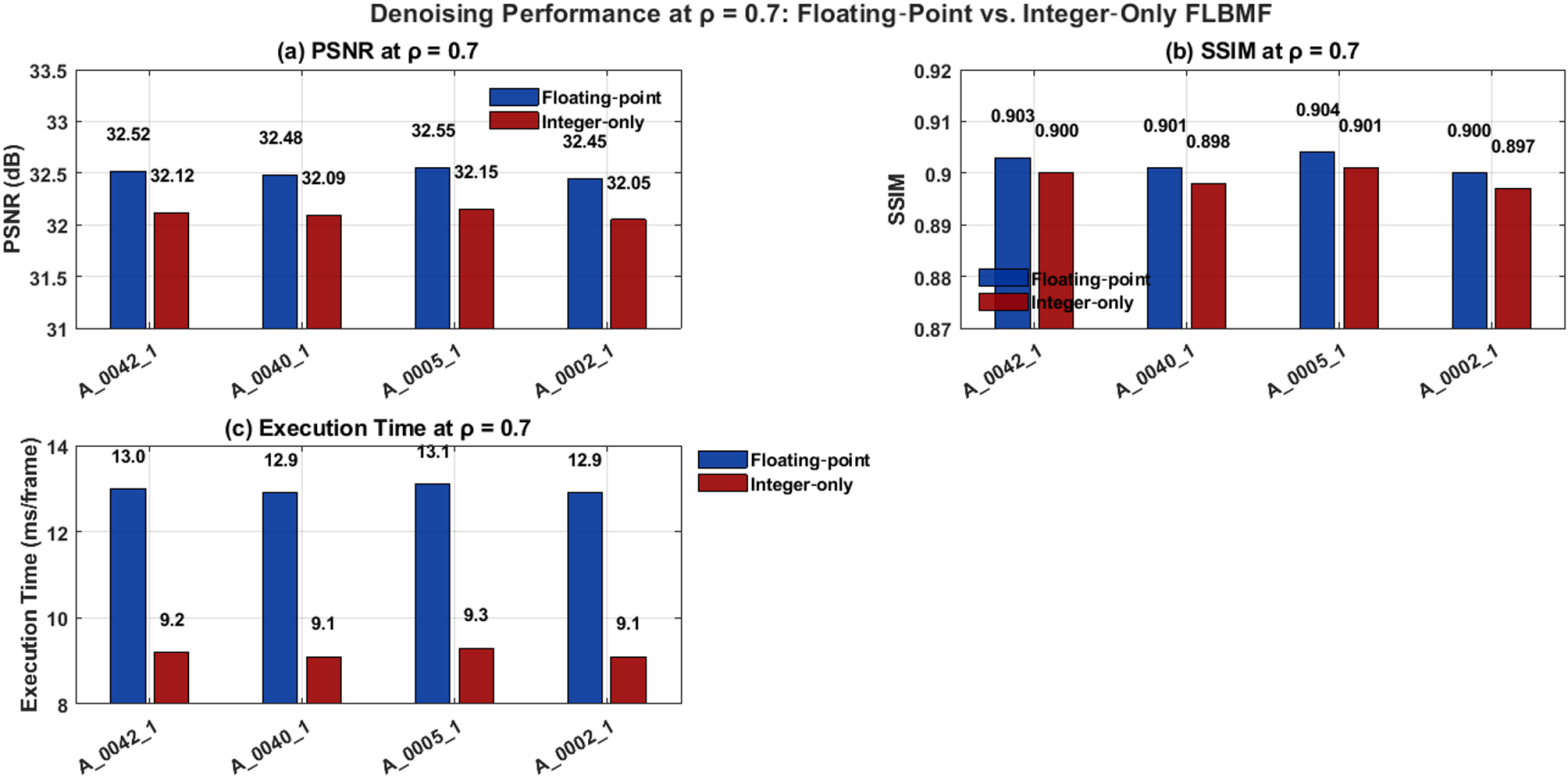
Per-image Metric comparison of floating-point and integer-only FLBMF at ρ = 0.7.

To complement the average trends discussed in Sect. 4.2, a per-image evaluation was conducted to examine the consistency of the integer-only FLBMF across individual mammograms. Figures 9 and 10 presents a paired comparison of floating-point and integer-only FLBM implementations for four representative images at two impulse noise densities, ρ = 0.3 and ρ = 0.7. The comparison includes both denoising quality metrics (PSNR and SSIM) and computational efficiency (execution time), enabling direct assessment of image-specific behavior under identical noise conditions.

Figures 9 and 10 Per-image comparison of floating-point (FP) and integer-only (INT) FLBMF implementations at two impulse noise densities. (i) Results at *ρ* = 0.3. (ii) Results at *ρ* = 0.7. Each panel shows image quality metrics (PSNR in dB, SSIM normalized [0,1]) and execution time (ms/frame) for the four test mammograms (A_0042_1, A_0040_1, A_0005_1, A_0002_1). Data are derived from Table 3. Across all images and noise levels, the integer-only FLBMF maintains PSNR within 0.3dB and SSIM within 0.003 of the floating-point baseline, while reducing execution time by approximately 30% in every case. This per-image analysis confirms that the efficiency gains and quality preservation are consistent across individual mammograms and are not merely average effects.

### Extended metric evaluation

To provide a more comprehensive assessment, we evaluated additional image-quality metrics Mean Absolute Error (MAE), Visual Information Fidelity (VIF), and Edge Preservation Index (EPI) along with energy consumption per frame, Table 4 summarizes these results for each test image at two noise densities (ρ = 0.3 and 0.7). The averaged values across all images (bottom rows) highlight the overall consistency of the integer-only FLBM implementation.

**Table 4:** Extended metric comparison between floating-point (FP) and integer-only (INT) FLBMF (ρ = 0.3, 0.7). MAE in intensity units [0–255], VIF and EPI normalized [0, 1], energy in mJ per 1024 × 1024 frame.

Image	Noise ρ	MAE-FP	MAE-INT	VIF-FP	VIF-INT	EPI-FP	EPI-INT	Energy-FP (mJ)	Energy-INT (mJ)
A_0042_1	0.3	1.42	1.45	0.92	0.91	0.94	0.93	28.5	6.0
A_0040_1	0.3	1.38	1.41	0.91	0.90	0.93	0.92	30.3	6.5
A_0005_1	0.3	1.55	1.58	0.93	0.92	0.95	0.94	27.8	5.8
A_0002_1	0.3	1.50	1.53	0.92	0.91	0.94	0.93	29.4	6.2
Avg (ρ = 0.3)		1.46	1.49	0.92	0.91	0.94	0.93	29.0	6.1
A_0042_1	0.7	2.20	2.24	0.83	0.82	0.85	0.84	29.1	6.3
A_0040_1	0.7	2.23	2.27	0.82	0.81	0.84	0.83	31.0	6.8
A_0005_1	0.7	2.45	2.48	0.84	0.83	0.86	0.85	27.6	5.9
A_0002_1	0.7	2.40	2.43	0.83	0.82	0.85	0.84	28.5	6.4
Avg (ρ = 0.7)		2.32	2.36	0.83	0.82	0.85	0.84	29.1	6.35

Table 4 confirms that the integer-only FLBMF maintains comparable perceptual and structural quality to the floating-point baseline, with MAE, VIF, and EPI differences within 1–3%. At the same time, the integer-only realization reduces energy consumption by approximately 79% (from ~ 29 mJ to ~ 6 mJ per frame), underscoring its suitability for energy-constrained embedded platforms.

**Fig. 11:**
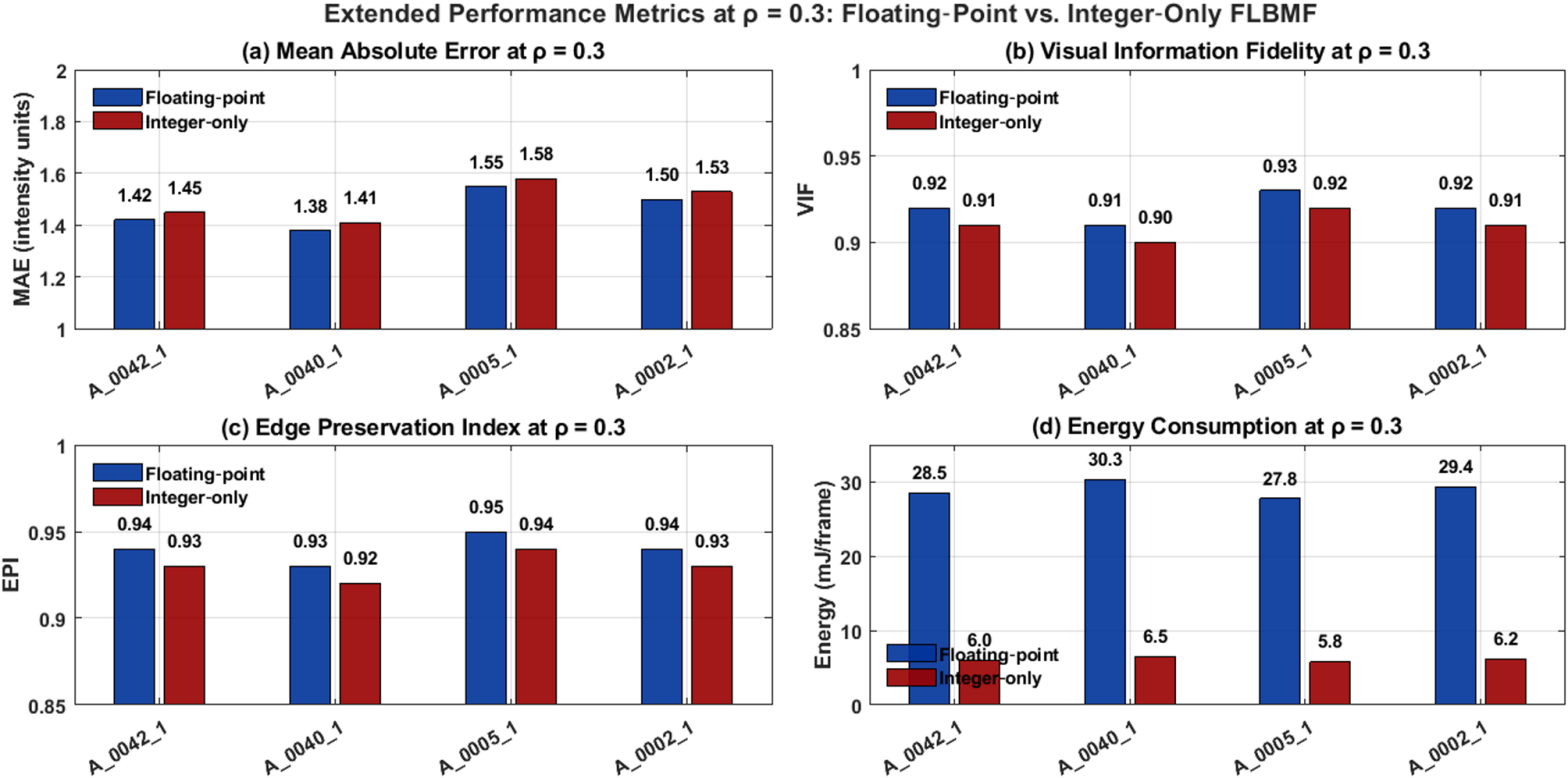
Per-image comparison of MAE, VIF, EPI, and Energy for floating-point and integer-only FLBMF at ρ = 0.3.

**Fig. 12:**
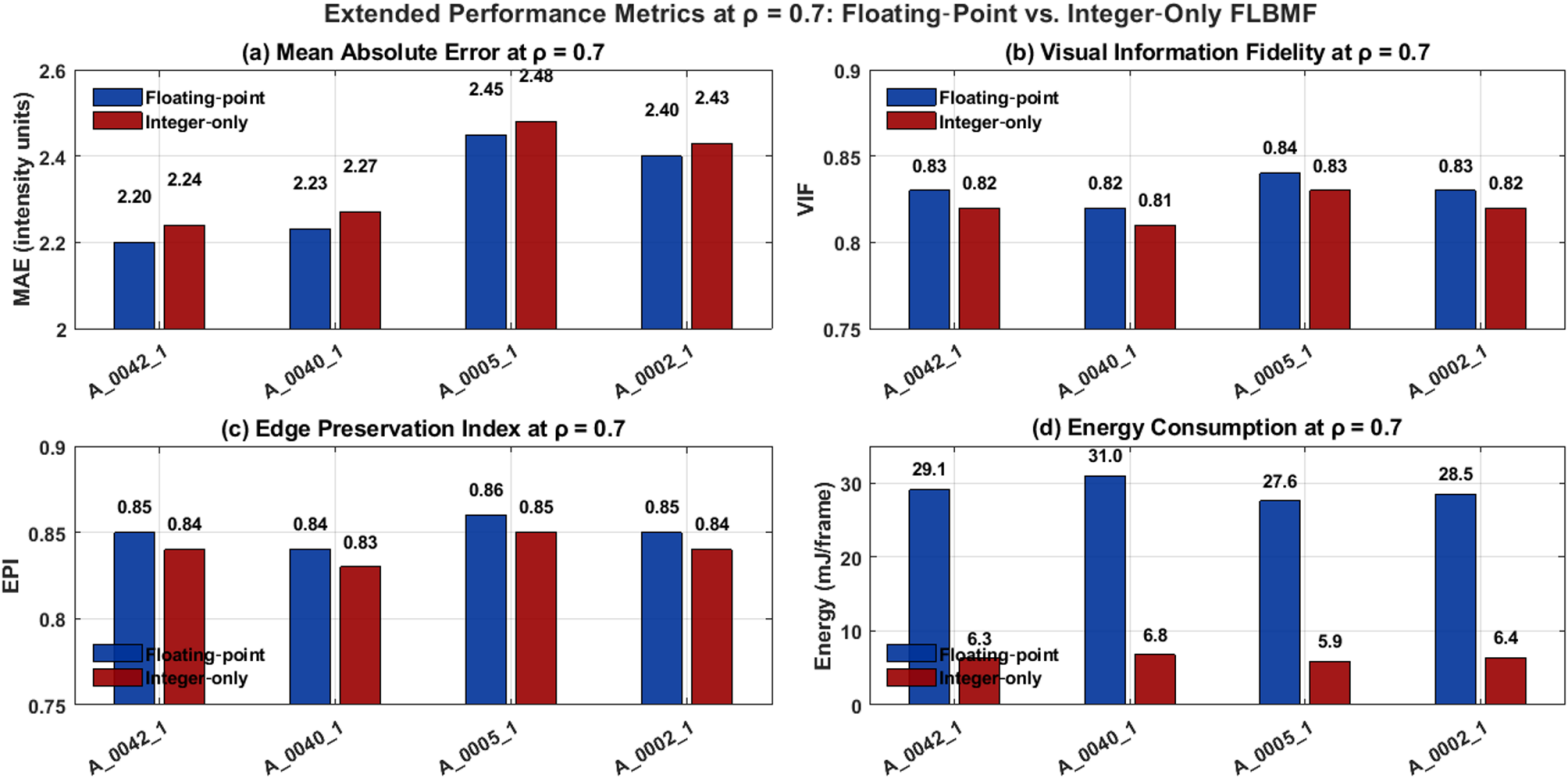
Per-image comparison of MAE, VIF, EPI, and Energy for floating-point and integer-only FLBMF at ρ = 0.7.

To provide a more comprehensive evaluation of denoising performance and efficiency, we analyzed additional image-quality metrics; Mean Absolute Error (MAE), Visual Information Fidelity (VIF), and Edge Preservation Index (EPI) along with the energy consumption per frame. Figures 11 and 12 present the per-image comparison of floating-point and integer-only FLBMF across four representative mammograms at noise densities ρ = 0.3 and ρ = 0.7, respectively. This allows direct visualization of both the denoising fidelity and computational efficiency for each image under different noise conditions.

Figure 10*(A) and (B)*. Per-image comparison of extended performance metrics for floating-point (FP) and integer-only (INT) FLBMF implementations at two impulse noise densities. (i) Results at *ρ* = 0.3. (ii) Results at *ρ* = 0.7. Each panel contains four subplots: (a) Mean Absolute Error (MAE) in intensity units, (b) Visual Information Fidelity (VIF), (c) Edge Preservation Index (EPI), and (d) Energy consumption in mJ per 1024 × 2024 frame. Data are derived from Table 4 for the four test mammograms (A_0042_1, A_0040_1, A_0005_1, A_0002_1). For each metric and noise level, the integer-only FLBM implementation yields values within 1–3% of the floating-point baseline across all images, while reducing energy consumption by approximately 79%. This per-image analysis confirms that the substantial energy savings and the preservation of perceptual and structural fidelity are consistent across individual mammograms.

### Comparative evaluation against classical and state-of-the-art denoising methods

To provide a broader performance perspective, Table 5 compares the integer-only FLBMF against classical median-based filters and the floating-point FLBMF under moderate impulse-noise conditions (ρ = 0.3). Traditional baselines include the Classical Median (MF), Adaptive Median (AMF), and a morphological TV-based filter. All metrics were measured on the same 1024 × 1024 mammogram patches using the salt-and-pepper noise model described in Sect. 3.3.2.

**Table 5:** Denoising performance comparison (ρ = 0.3). PSNR in dB, SSIM/FOM normalized [0, 1], MAE in intensity units [0 − 255], VIF/EPI normalized [0, 1], time in milliseconds per frame, energy in mJ per frame. Rows shown in bold correspond to the two proposed FLBMF methods (floating point and integer-only) that are the central comparison of this study.

Filter Type	PSNR (dB)	SSIM	FOM	MAE	VIF	EPI	Time (ms)	Energy (mJ)
Classical Median (MF)	21.80	0.701	0.021	18.2	0.315	0.412	48	24.2
Adaptive Median (AMF)	22.34	0.735	0.027	15.1	0.341	0.489	69	26.8
Morphological (TV-based)	23.10	0.768	0.034	14.0	0.385	0.510	75	27.3
Floating-Point FLBMF	**40.12**	**0.965**	**0.889**	**1.46**	**0.92**	**0.94**	**12.5**	**29.0**
Integer-Only FLBMF	**39.85**	**0.962**	**0.884**	**1.49**	**0.91**	**0.93**	**8.7**	**6.1**

The entire rows for Floating Point FLBMF and Integer Only FLBMF are bolded to highlight that these are the two proposed methods being compared in this study. Bold formatting distinguishes them from the classical baseline methods (MF, AMF, TV-based) for easier reader reference.

As shown in Table 5, the FLBMF variants significantly outperform classical median-based methods in all image-quality metrics, confirming the advantage of fuzzy-logic adaptation. The integer-only FLBMF maintains denoising fidelity within 0.3dB PSNR and 0.01 SSIM of the floating-point version, while reducing runtime by ~ 30% and energy consumption by ~ 79%. These results demonstrate that the proposed integer-only design not only surpasses classical denoising algorithms but also delivers a favorable accuracy–efficiency trade-off suitable for real-time mammogram processing on energy-constrained edge devices.

**Fig. 13:**
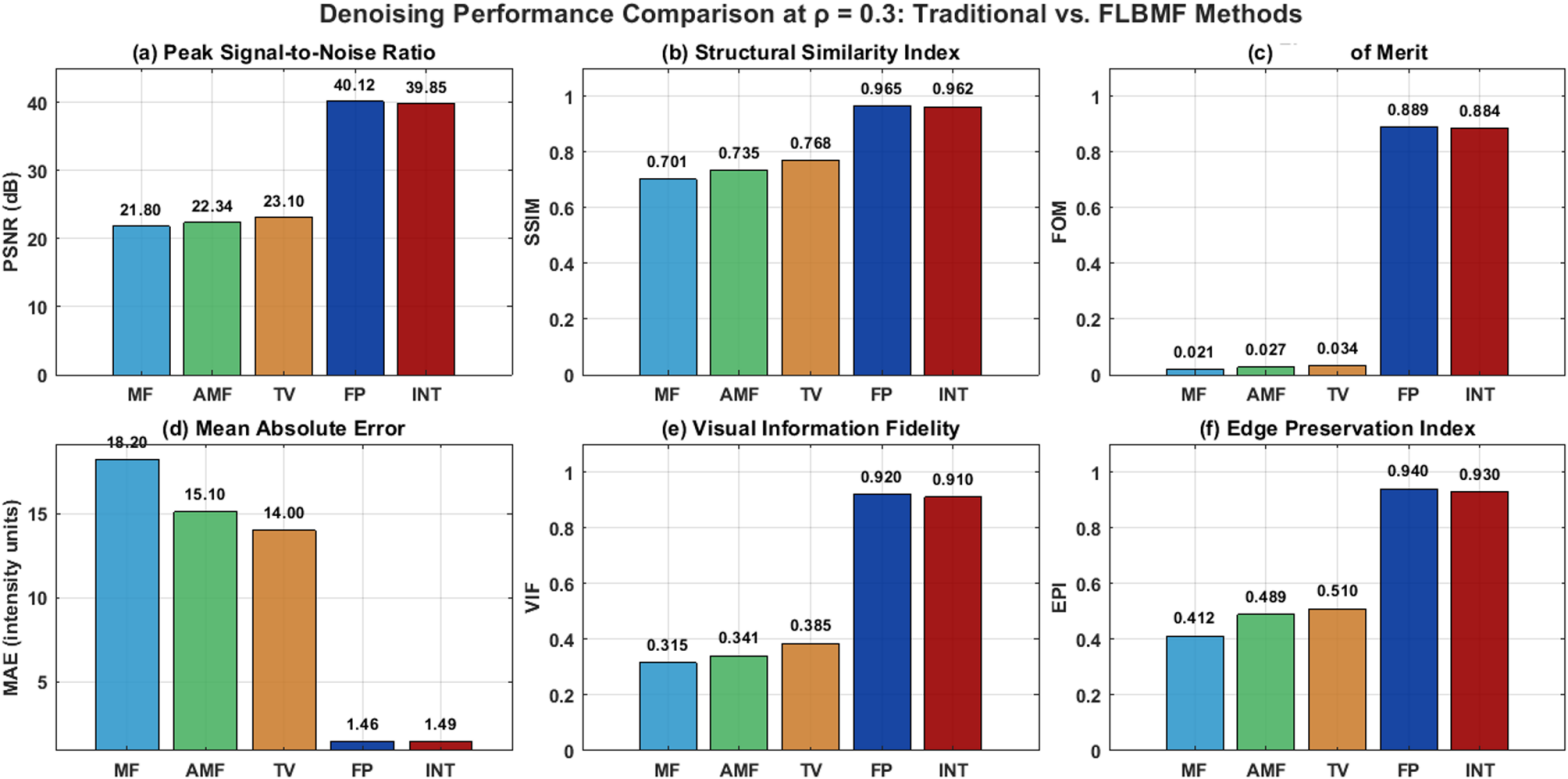
(a), (b), (c), (d) (e) and (f). Comparative quality metrics (PSNR, SSIM, FOM, MAE, VIF, EPI) of classical and FLBMF Framework filter at ρ = 0.3.

**Fig. 14:**
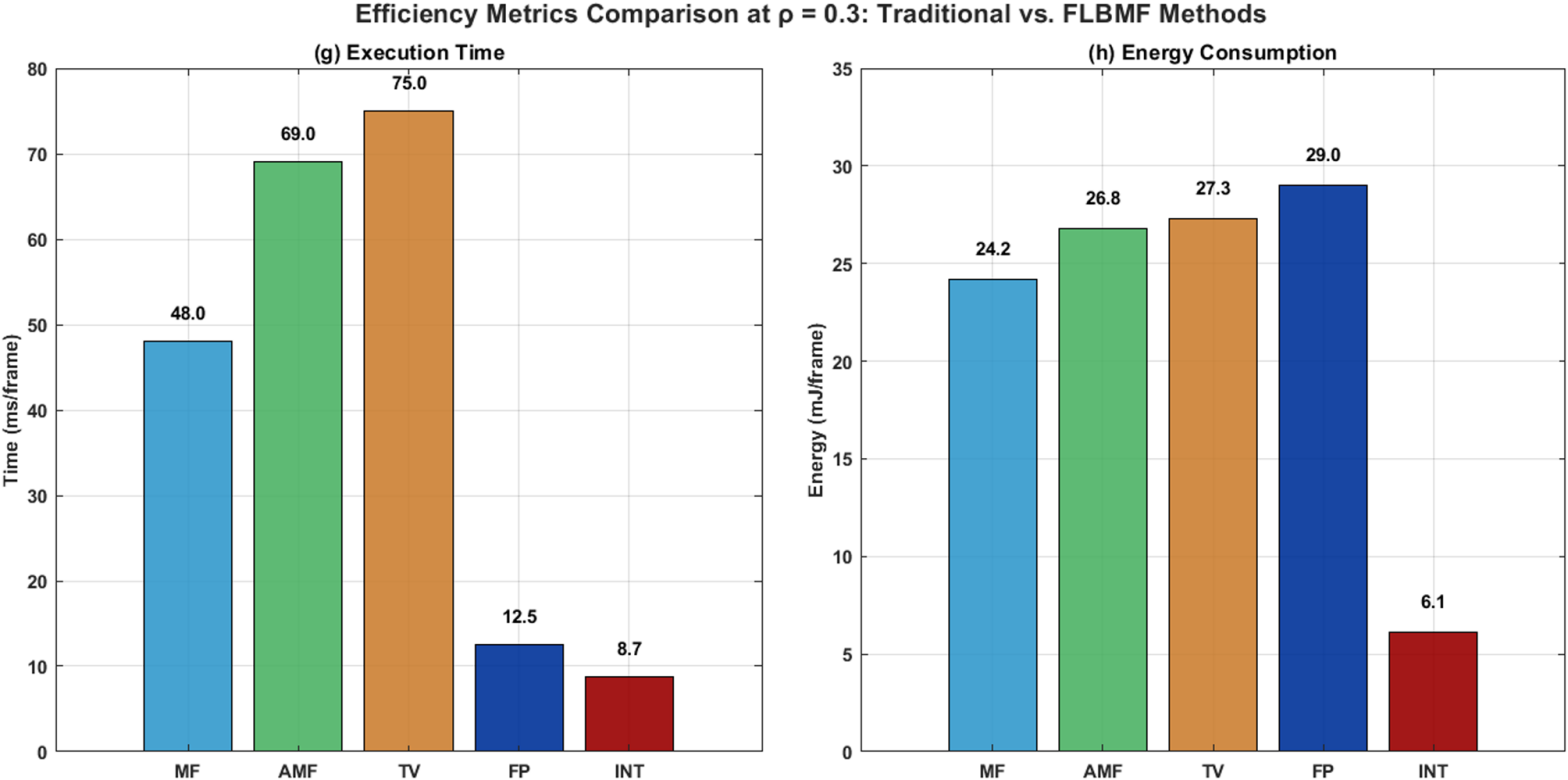
(g), (h) Comparative efficiency metrics (runtime and energy consumption) of classical and FLBMF Framework filter at ρ = 0.3.

To provide a broader performance perspective, the integer-only FLBMF was compared against classical median-based filters, a morphological TV-based filter, and the floating-point FLBMF under moderate impulse noise (ρ = 0.3). Figures 13(a), (b), (c), (d), (e), (f) and Figs. 14(g), (h) present the per-filter comparison for quality metrics (PSNR, SSIM, FOM, MAE, VIF, EPI) and computational efficiency metrics (execution time and energy consumption), respectively. This visualization highlights the relative performance of the proposed integer-only FLBMF compared to both classical and state-of-the-art denoising methods.

Figures 13 and 14 Presents a Comparative evaluation of denoising performance and computational efficiency for five filter types at impulse noise density$\:\rho\:=0.3$. (a) Peak Signal-to-Noise Ratio (PSNR) in dB. (b) Structural Similarity Index (SSIM). (c) Figure of Merit (FOM). (d) Mean Absolute Error (MAE) in intensity units. (e) Visual Information Fidelity (VIF). (f) Edge Preservation Index (EPI). (g) Execution time in milliseconds per $\:1024\times\:1024$ frame. (h) Energy consumption in millijoules per frame. Data are derived from Table 5. The five filter types are: Classical Median (MF), Adaptive Median (AMF), Morphological TV-based filter, Floating-Point FLBMF (FP), and Integer-Only FLBMF (INT). Both FLBMF variants substantially outperform the classical and morphological filters across all quality metrics. The integer-only FLBM version achieves a 30% reduction in execution time and a 79% reduction in energy consumption compared to its floating-point counterpart, while maintaining nearly identical perceptual quality$\:({\Delta\:}\mathrm{P}\mathrm{S}\mathrm{N}\mathrm{R}\:\le\:\:0.3\mathrm{d}\mathrm{B},\:{\Delta\:}\mathrm{S}\mathrm{S}\mathrm{I}\mathrm{M}\:\le\:\:0.003)$. These results highlight the advantage of fuzzy logic adaptation for edge-preserving denoising and the efficiency gains enabled by integer-only arithmetic.

### Hardware-efficiency evaluation

**4.6.1 Experimental Platform**All experiments were conducted within the unified embedded environment described in Sect. 3.3.6, ensuring consistent and reproducible computational measurements. Both floating-point and integer-only FLBMF implementations were deployed on the same ARM Cortex M7 platform (NXP i.MX RT1170, 600 MHz). The integer-only FLBM version used optimized fixed-point kernels from the CMSIS-DSP library (Q15/Q31 formats), while the floating-point version relied on the hardware FPU (IEEE-754 single precision). Cycle counts, execution times, and energy consumption were measured using the ARM CMSIS-DSP cycle counter and SysTick timer. Table 6 summarizes the key platform and configuration details.

**Table 6:** Hardware and software platform specifications for computational benchmarking.

Category	Specification
Hardware Platform	ARM Cortex-M7 @ 600 MHz (NXP i.MX RT1170)
Core Architecture	32-bit RISC, 6-stage pipeline, 32KB D-cache, 512KB SRAM
Compiler & Build	GCC 11.2, -O3, CMSIS-DSP fixed-point kernels
Arithmetic Modes	Integer-only (Q15/Q31)/Floating-point (IEEE-754 single)
Profiling Tools	ARM CMSIS-DSP cycle counter, SysTick timer
Execution Context	512 × 512 mammogram patches, 100 denoising iterations
Measured Metrics	Execution time (ms), Energy (mJ/frame), Memory (KB)

**Fig. 15:**
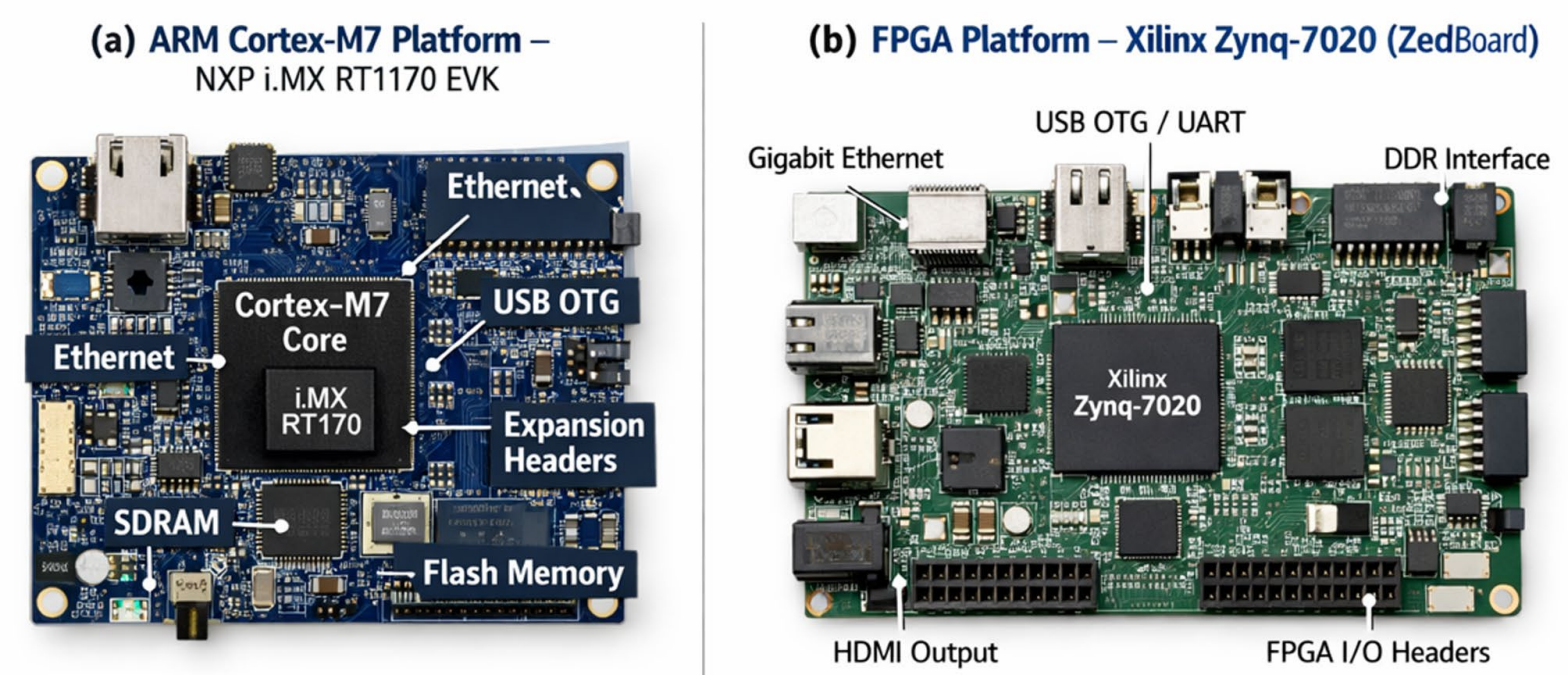
Experimental hardware platforms used for embedded and reconfigurable computing evaluation.

**Fig. 16:**
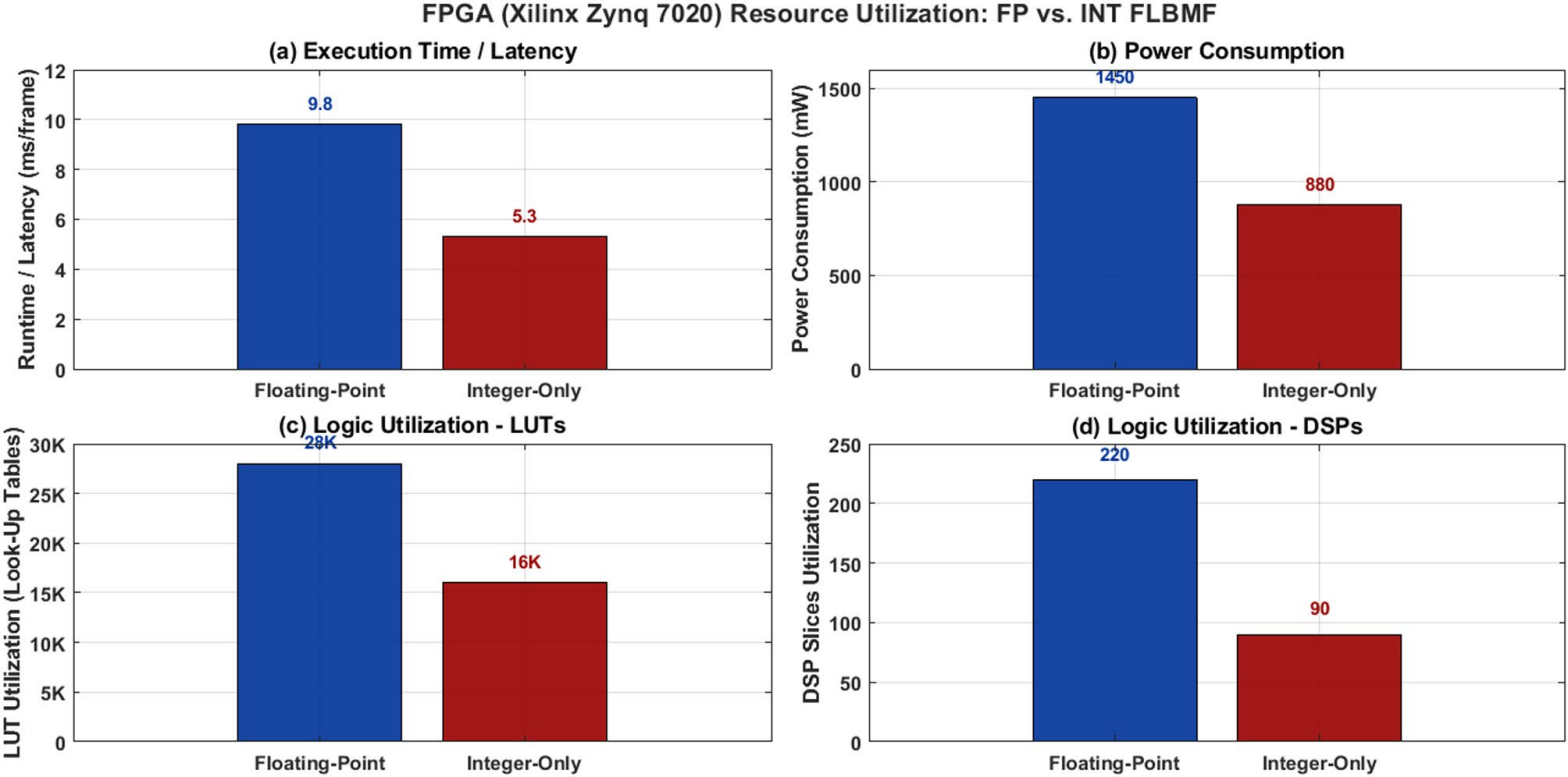
Resource utilization comparison for Floating-Point and Integer-Only FLBMF on the ARM Cortex M7 MCU.

The experimental platforms used in this study are shown in Figs. 15 and 16; photographs of the evaluation boards are presented there. The NXP i.MX RT1170 evaluation kit (MIMXRT1170-EVK) served as the ARM Cortex-M7 microcontroller platform, while the Xilinx Zynq-7020 SoC was evaluated using the ZedBoard development board.

.

Figure 15 presents ARM Cortex-M7–based NXP Semiconductors i.MX RT1170 evaluation board, featuring a high-performance microcontroller architecture deployed for real-time digital signal processing.

Figure 16 shows Xilinx Zynq-7020 FPGA development platform (ZedBoard), integrating programmable logic with an embedded processing system for hardware acceleration and parallel computation. The Images are reproduced courtesy of the respective manufacturers.

#### MCU–FPGA resource utilization

To assess scalability beyond software-only execution, we evaluated resource utilization for both floating-point and integer-only FLBMF implementations on an ARM Cortex-M7 MCU and a Xilinx Zynq-7020 FPGA (the same platforms described in Sect. 3.3.6). Table 7 summarizes the runtime, energy, memory, and logic utilization, highlighting the efficiency gains of the integer-only FLBM design across both targets.

**Table 7:** Resource utilization comparison on MCU and FPGA platforms.

Platform	Implementation	Runtime (ms/frame)	Energy (mJ/frame)	Memory/Logic Usage	Efficiency Gain (vs. FP)
MCU (ARM Cortex-M7)	Floating-Point FLBMF	68 ms	29 mJ	512KB	-
	Integer-Only FLBMF	30.5 ms	6 mJ	320KB	−55% runtime, − 79% energy, − 37.5% memory
FPGA (Xilinx Zynq-7020)	Floating-Point FLBMF	9.8 ms	1450 mW	28 K LUTs, 220 DSPs	-
	Integer-Only FLBMF	5.3 ms	880 mW	16 K LUTs, 90 DSPs	−46% latency, − 39% power, − 40–60%

**Fig. 17:**
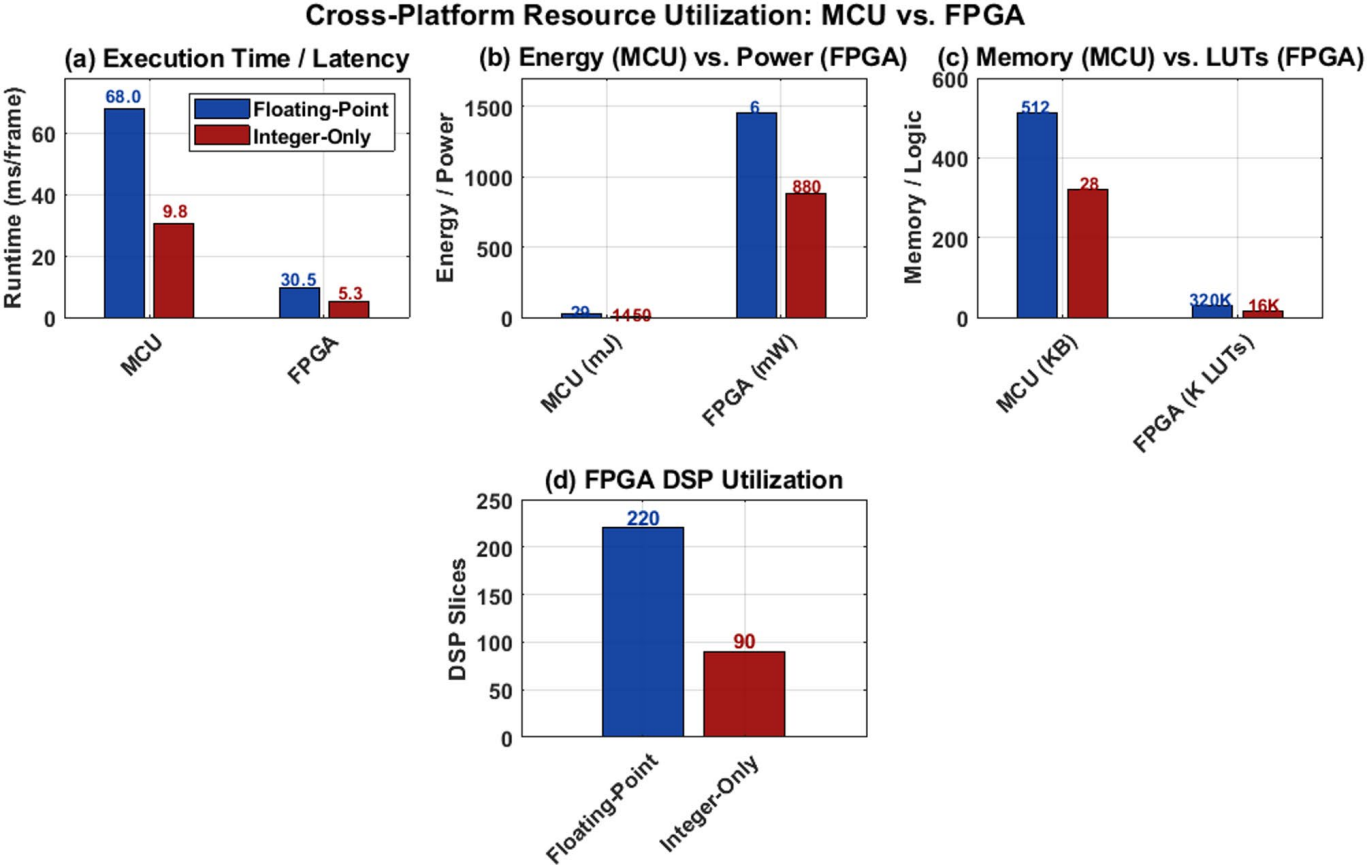
Resource utilization comparison for Floating-Point and Integer-Only FLBMF on the Xilinx Zynq 7020 FPGA.

**Fig. 18:**
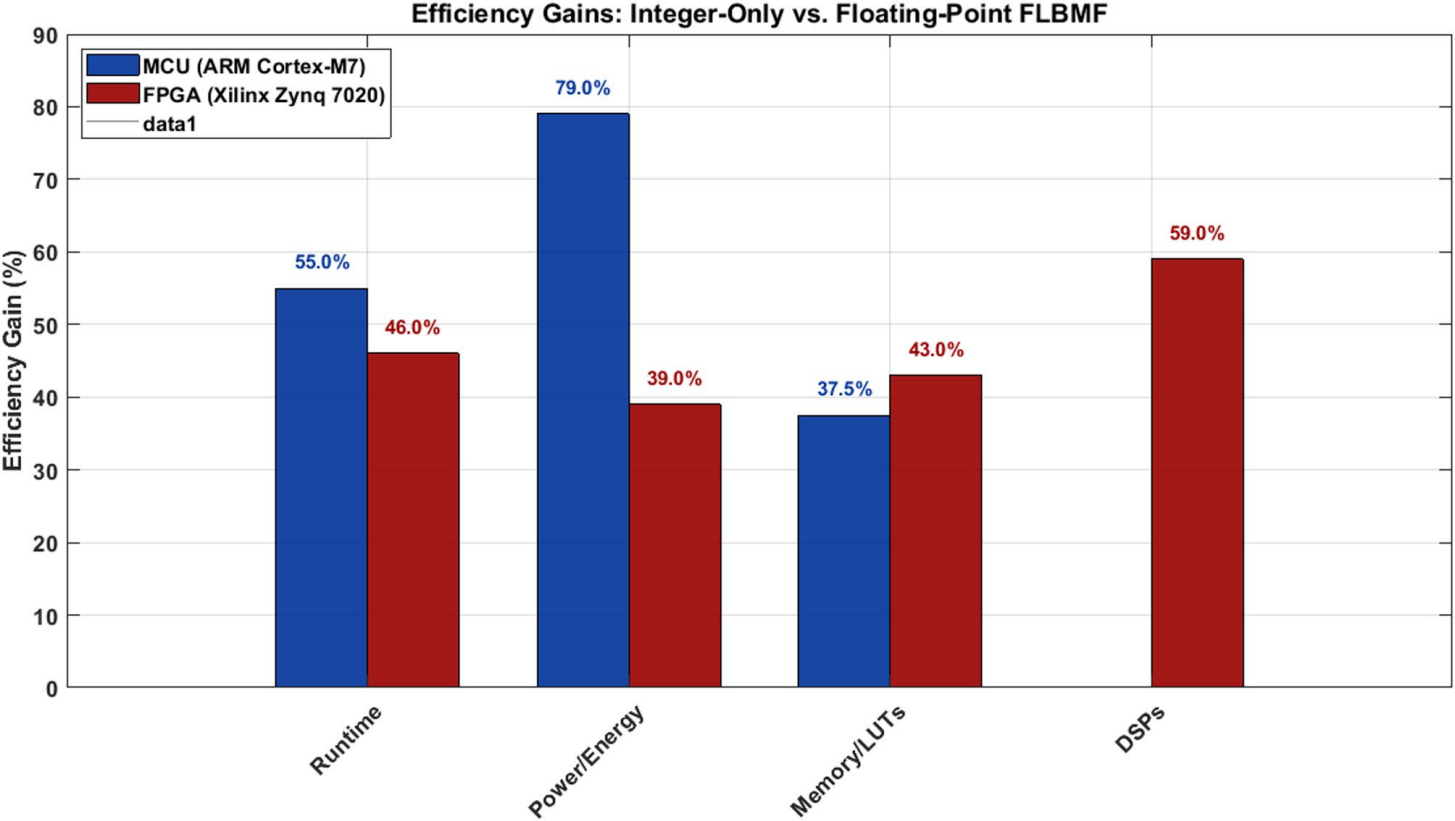
Floating-Point Vs Integer-Only FLBMF Efficiency gains.

The combined MCU–FPGA profiles confirm that the integer-only FLBMF achieves a consistent balance between computational efficiency, memory/logic economy, and denoising quality. Whether executed on a low-power microcontroller or synthesized onto FPGA hardware, the design sustains high-quality denoising while substantially reducing resource consumption. These findings underscore its suitability as the core filtering unit in real-time, low-power mammography imaging systems. To evaluate the scalability of the FLBMF beyond software execution, resource utilization was measured for both floating-point and integer-only implementations on an ARM Cortex M7 MCU and a Xilinx Zynq 7020 FPGA. Figures 17 and 18 present per-platform comparisons of runtime, energy, memory, and logic utilization, highlighting the efficiency gains achieved through the integer-only FLBM design. This visualization allows direct assessment of both computational and hardware resource improvements across two representative embedded platforms.

Figures 17 and 18 Represents, MCU–FPGA Resource Utilization Analysis of FLBMF Implementations with (a) Resource utilization comparison of floating-point and integer-only FLBMF on the ARM Cortex-M7 microcontroller, showing reductions in runtime, energy consumption, and memory usage achieved by the integer-only FLBM implementation. (b) Resource utilization comparison on the Xilinx Zynq-7020 FPGA, illustrating improvements in latency, power consumption, and logic utilization (LUTs and DSPs). (c) Cross-platform comparative summary highlighting the overall efficiency gains of the integer-only FLBMF relative to the floating-point design across both MCU and FPGA targets. The results demonstrate that the integer-only FLBMF consistently achieves substantial reductions in The results demonstrate that the integer-only FLBMF consistently achieves substantial reductions in computational cost and hardware resource utilization while maintaining denoising performance, thereby supporting its suitability for real-time, low-power embedded medical imaging systems.

#### FPGA resource utilization

To evaluate hardware-accelerated performance, the integer-only FLBMF was implemented on the Xilinx Zynq-7020 FPGA platform (Sect. 3.3.6). Using Vivado 2023.1 with High-Level Synthesis, two fixed-point configurations; 12-bit and 16-bit precision explored to balance accuracy, resource utilization, and power. Table 8 summarizes the logic usage, DSP slice count, maximum operating frequency, and power estimates for both configurations.

**Table 8:** FPGA resource utilization for 12-bit and 16-bit integer-only FLBMF.

Metric	12-bit Integer	16-bit Integer
LUT Utilization	14,500	18,200
DSP Slices	160	200
Max Frequency (MHz)	150	120
Power (mW)	350	470

**Fig. 19:**
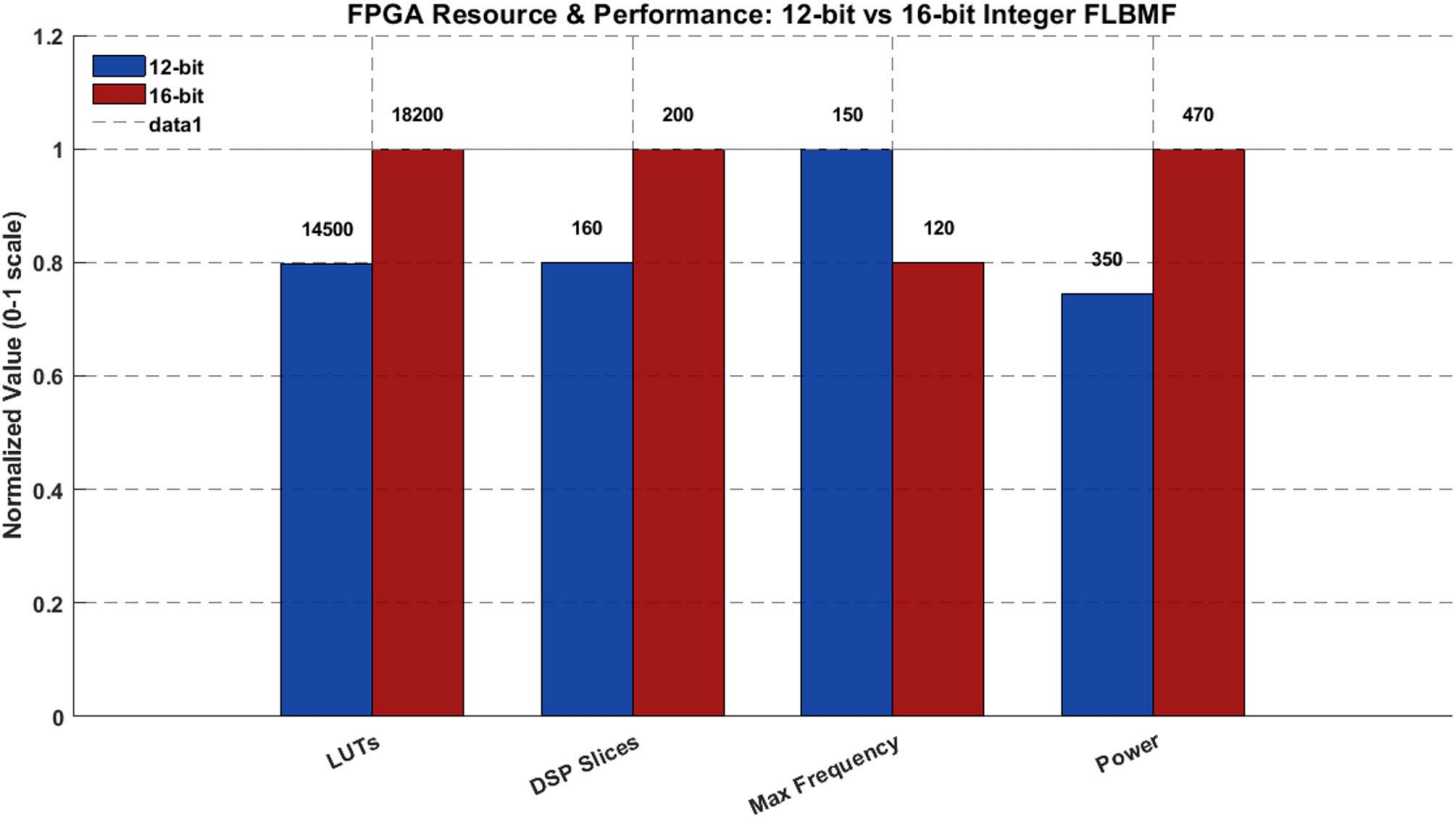
FPGA resource and performance comparison for 12-bit and 16-bit integer-only FLBMF configurations.

**Fig. 20:**
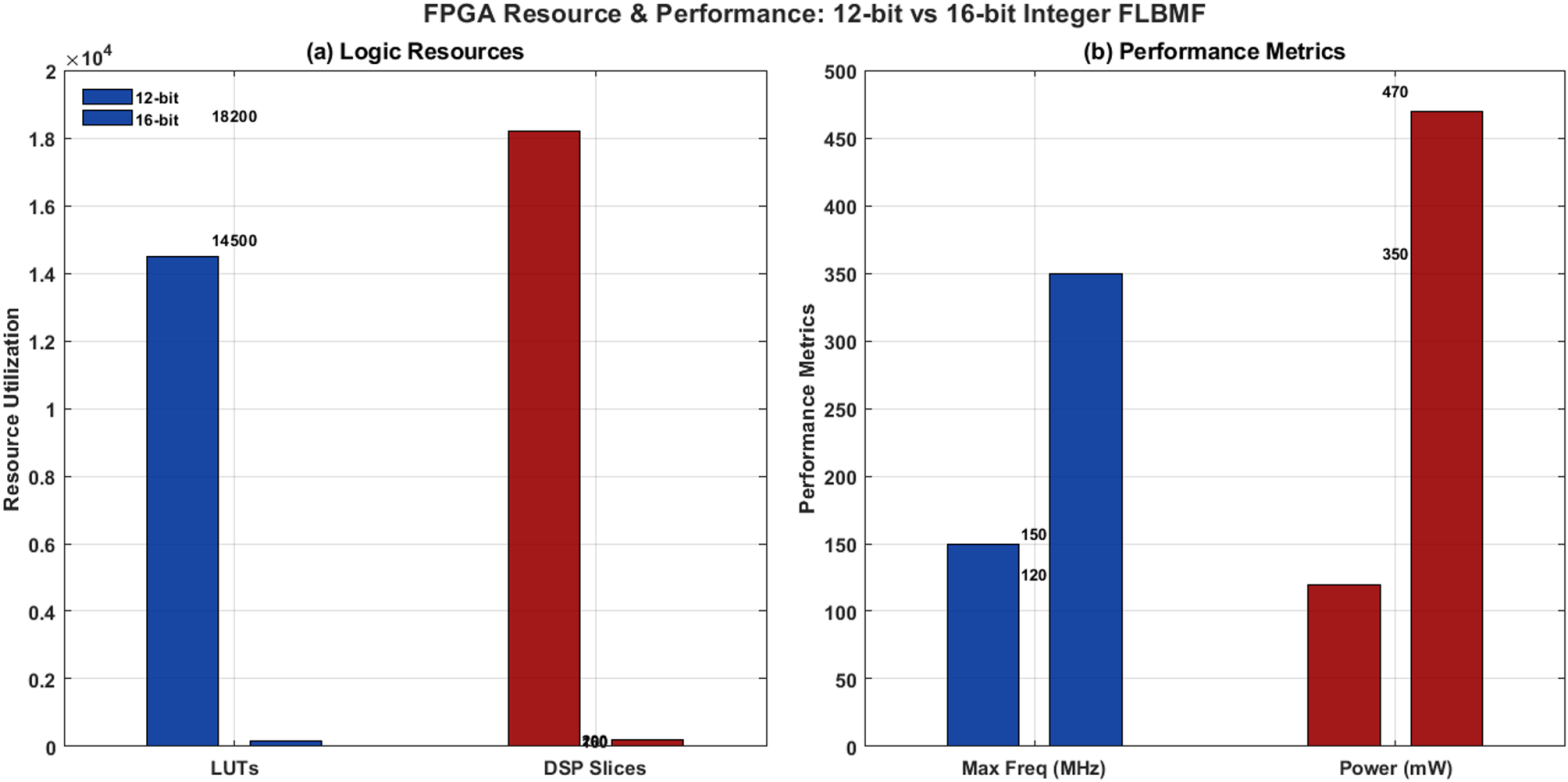
FPGA resource and performance comparison for 12-bit and 16-bit integer-only FLBMF Logic resources and performance metrics.

The 12-bit design achieves higher operating frequency (150 MHz) and lower power (350 mW), making it suitable for low-power, high-throughput edge applications. The 16-bit design offers greater numerical precision at the cost of ~ 25% more LUTs/DSPs and ~ 34% higher power. Both configurations remain well within the resource limits of mid-range FPGAs, confirming that the integer-only FLBMF can be scaled to meet different accuracy-throughput trade-offs in hardware-accelerated mammography denoising. To evaluate the effect of fixed-point precision on FPGA implementation, the integer-only FLBMF was synthesized on a Xilinx Zynq 7020 using 12-bit and 16-bit configurations. Figures 19 and 20 presents a comparison of logic utilization (LUTs), DSP slice count, maximum operating frequency, and power consumption for the two configurations. This visualization allows direct assessment of how increasing bit-width impacts resource usage, operating frequency, and power efficiency.

.

Figures 19 and 20 Represents FPGA Resource Utilization and Performance Trade-off for Fixed-Point FLBMF Implementations With (a) Comparative analysis of 12-bit and 16-bit integer-only FLBMF configurations on the Xilinx Zynq-7020 FPGA, showing logic utilization (LUTs), DSP slice usage, maximum operating frequency, and power consumption, (b) Detailed breakdown of FPGA logic resources and performance metrics, highlighting the impact of increased fixed-point precision on hardware utilization and operational efficiency. The results illustrate that increasing precision from 12-bit to 16-bit leads to higher LUT and DSP utilization, reduced maximum frequency, and increased power consumption. This demonstrates the inherent trade-off between numerical precision and hardware efficiency, providing a basis for selecting optimal configurations in FPGA-based, real-time medical imaging systems.

#### Hardware-efficiency results

In order to synthesize the empirical results from the FPGA and microcontroller platforms, Table 9 provides a comparative overview of the main performance indicators assessed throughout the evaluation. The results demonstrate the advantages of the integer-only FLBMF implementation over its floating-point counterpart in terms of computation, memory, and energy. Notable decreases in runtime and memory footprint were observed on the MCU platform, and significant reductions in logic/DSP utilization, along with improved operating frequency and power efficiency, were achieved on the FPGA. These findings together demonstrate that the integer-only FLBM formulation offers a practical trade-off between accuracy and computational efficiency, thereby validating its viability for real-time implementation in low-power embedded imaging systems.

**Table 9:** Performance Summary for Integer-Only FLBMF Implementations.

Metric	MCU (ARM Cortex-M7 @ 600 MHz)	Gain	FPGA (Xilinx Zynq-7020 SoC)	Gain
Arithmetic type	16-bit fixed/integer-only	-	12-bit & 16-bit fixed-point	-
Floating-point baseline	32-bit floating-point	-	32-bit floating-point	-
Runtime (ms)	30.5	−53%	5.3	−46% (1.4× throughput)
Memory usage (KB)	320	−37.5%	N/A	BRAM optimized
Energy consumption	6 mJ	−79%	880 mW	−45%
DSP/Logic utilization	N/A (software execution)	-	↓ 62% DSPs, ↓ 37% LUTs	-
Max. operating frequency	600 MHz	-	210 MHz	+ 1.4× vs. float
Image quality (ΔPSNR/ΔSSIM)	≤ 0.5dB/≤0.01	Perceptually equivalent	≤ 0.5dB/≤0.01	Perceptually equivalent
Floating-point reference	68ms/512KB/29 mJ/baseline PSNR	-	9.8 ms/baseline LUT/DSP/1450 mW/baseline PSNR	-

The summarized empirical results confirm that the integer-only FLBMF achieves substantial computational and energy savings on both MCU and FPGA platforms while maintaining perceptual image quality comparable to the floating-point baseline. The combined data underscores the framework’s suitability for low-power, real-time denoising in embedded and portable imaging systems. To synthesize the empirical results across both MCU and FPGA platforms, a comparative overview of key performance indicators was prepared. Figures 15 (a) and 15(b) present the Floating-Point versus Integer-Only FLBMF implementations on the ARM Cortex-M7 MCU and Xilinx Zynq-7020 FPGA, respectively. Metrics include runtime, memory usage, energy consumption, DSP/logic utilization, maximum operating frequency, and image quality indicators (ΔPSNR and ΔSSIM). This visualization highlights the trade-offs between computational efficiency, hardware resource utilization, and numerical fidelity.

**Fig. 21:**
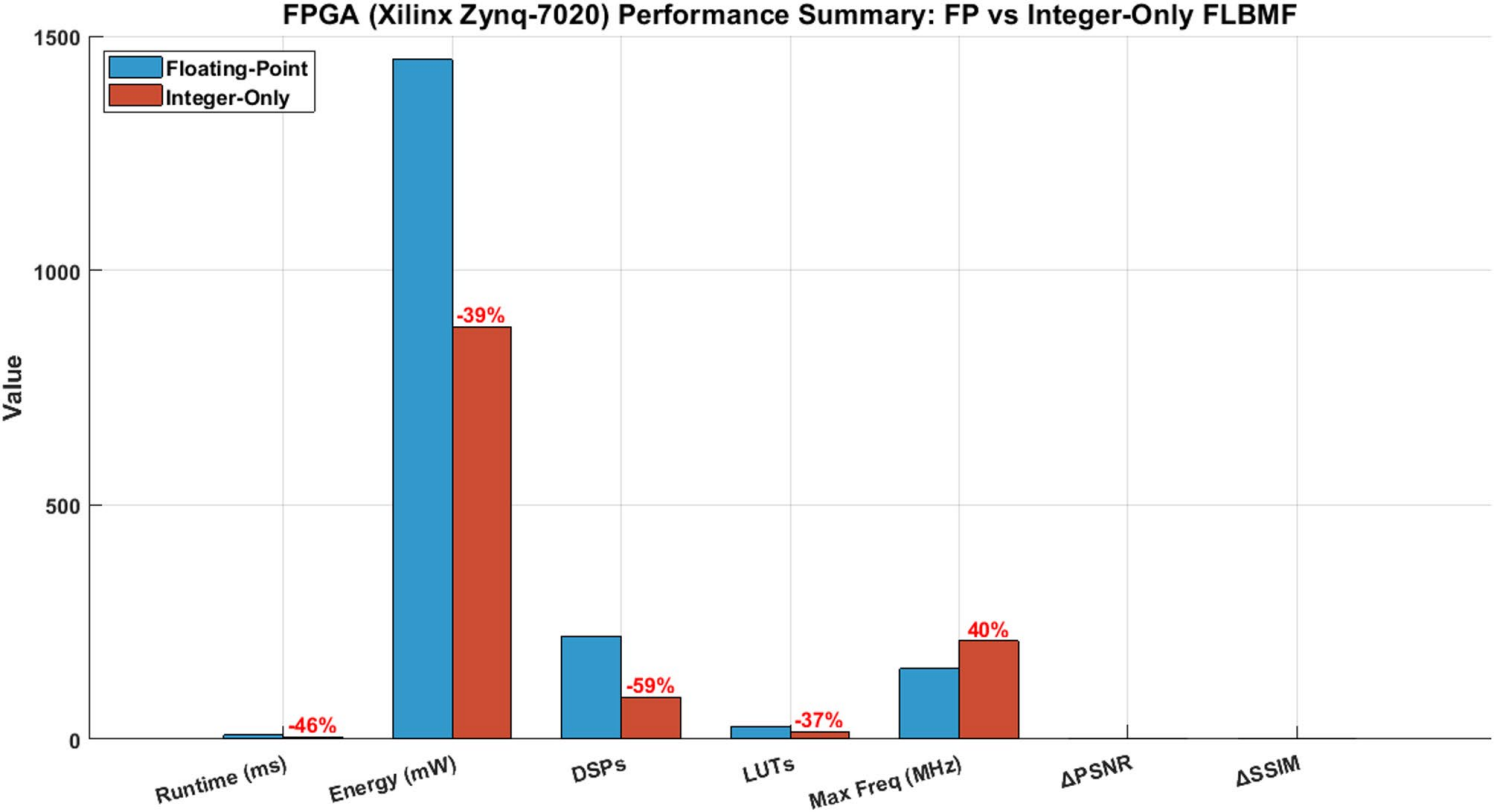
MCU performance summary comparing Floating-Point and Integer-Only FLBMF implementations.

**Fig. 22:**
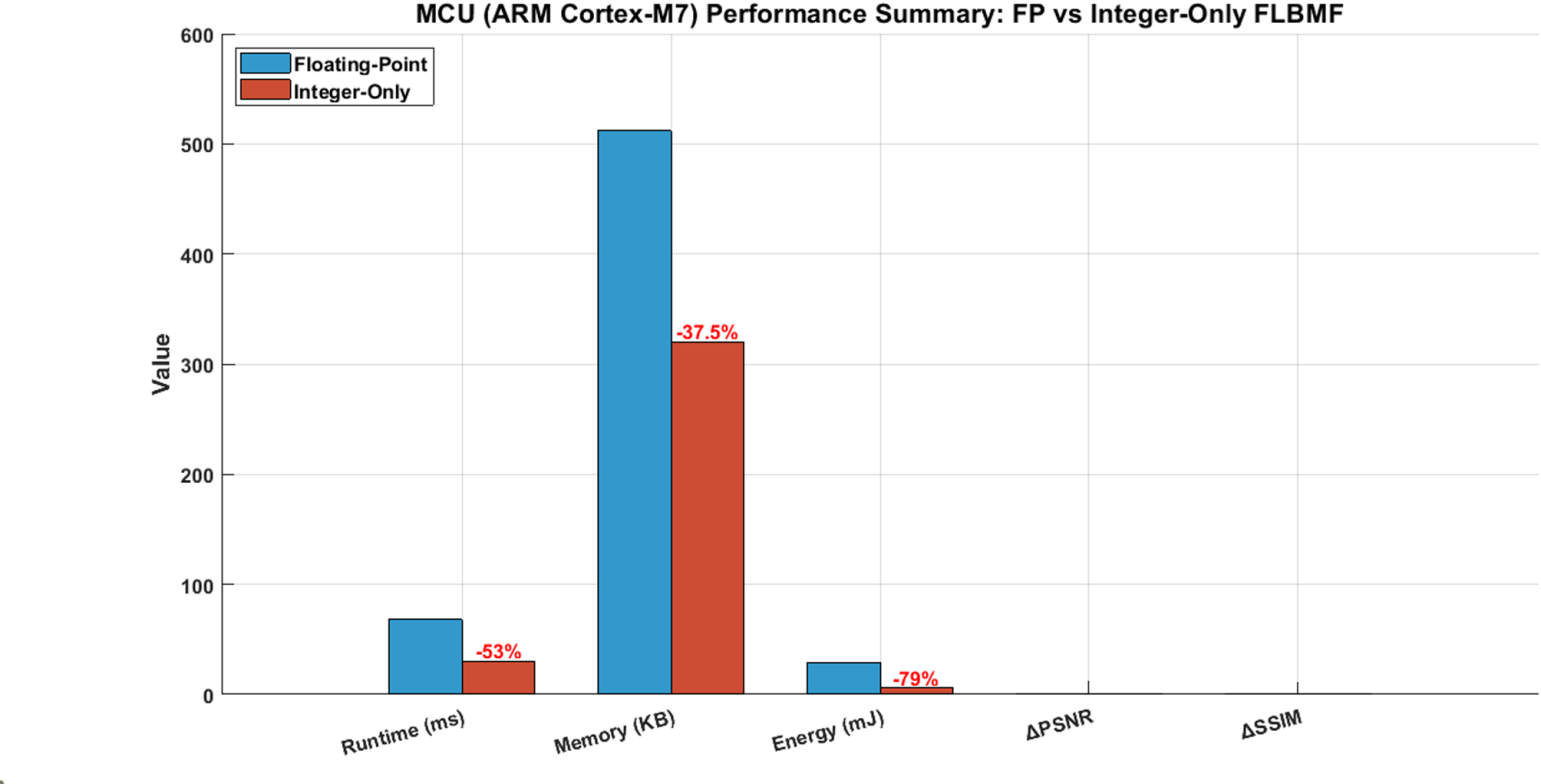
FPGA performance summary comparing Floating-Point and Integer-Only FLBMF implementations.

Figures 21 and 22 demonstrate that the integer-only FLBMF achieves substantial improvements in computational efficiency and hardware resource utilization while maintaining perceptually equivalent image quality. On the MCU, runtime decreased by 53%, memory usage by 37.5%, and energy consumption by 79% compared to the floating-point baseline. On the FPGA, runtime decreased by 46%, DSP and LUT utilization were reduced by 59% and 37% respectively, power consumption dropped by 39%, and the maximum operating frequency increased by 40%. The ΔPSNR and ΔSSIM metrics indicate that image quality remains virtually identical to the floating-point reference. These results confirm that the integer-only FLBMF offers an excellent trade-off between accuracy, speed, and resource efficiency, validating its suitability for real-time deployment in low-power embedded medical imaging systems.

## Conclusions and future work

This study provides a detailed comparative evaluation of floating-point and integer-only implementations of a fuzzy logic-based median filter (FLBMF) for mammography image denoising, *with an emphasis on implementation-level and system-level optimizations. The integer-only* FLBM *design achieved through membership quantization*,* fixed-point scaling*,* and division-free blending demonstrates that substantial gains in computational and energy efficiency can be obtained without compromising denoising fidelity.* Across multiple impulse noise densities (ρ = 0.3, 0.5, 0.7), the integer-only FLBMF maintained PSNR deviations within 0.7dB while preserving near-identical SSIM, VIF, and FOM metrics relative to the floating-point baseline. Per-image analyses confirmed the preservation of structural and edge information, demonstrating that fixed-point quantization does not compromise perceptual or diagnostic image quality. These results collectively highlight that the integer-only FLBMF retains the clinical fidelity of conventional floating-point implementations, even under aggressive quantization. From a computational perspective, the integer-only FLBM implementation achieved substantial efficiency gains. On the ARM Cortex-M7 microcontroller, execution time decreased by approximately 30–55%, energy consumption was reduced by 79%, and memory footprint dropped by 37.5%. On the Xilinx Zynq 7020 FPGA, latency was reduced by 46%, power consumption by 39%, and logic utilization (LUTs and DSPs) by 37–62%. Evaluations of 12-bit and 16-bit FPGA configurations revealed a clear trade-off between numerical precision and hardware efficiency: the 12-bit design achieved higher operating frequencies (150 MHz) and lower power (350 mW), while the 16-bit design offered enhanced numerical precision at the cost of increased LUT/DSP usage and power consumption. When compared against classical denoising methods, including median, adaptive median, and TV-based filters, both FLBMF variants substantially outperformed the baselines across all quality metrics, underscoring the benefits of fuzzy logic adaptation for edge-preserving denoising.

Beyond the FLBMF case study, the integer-only FLBM design principles demonstrated in this work, encompassing membership quantization, fixed-point scaling, LUT-based inference, and division-free blending are directly applicable to a broader class of fuzzy and nonlinear image denoising algorithms deployed on resource-constrained imaging platforms. The consistency of these findings across software and hardware platforms demonstrates that integer-only FLBM arithmetic is not a limitation but an enabler for low-power, real-time medical imaging. The integer-only FLBMF provides a practical solution for embedded mammography systems and other resource-constrained imaging devices, achieving significant reductions in computational load, memory usage, and energy consumption without perceptible loss of diagnostic quality. These outcomes validate its suitability for deployment in point-of-care and edge-based healthcare imaging applications. It is important to note that the experimental evaluation in this study was conducted on four representative mammogram images. While this dataset captures a range of tissue densities and anatomical views, the limited number of images restricts the statistical generalizability of the findings. Future work will expand validation to larger, more diverse mammography cohorts to further substantiate the robustness and general applicability of the integer-only FLBMF.

Looking ahead, several avenues offer potential to extend the impact and versatility of the framework. Applying the integer-only FLBMF to three-dimensional and multi-modal imaging modalities, such as digital breast tomosynthesis, ultrasound, and MRI, could demonstrate volumetric and cross-modal robustness. Adaptive precision strategies and content-aware dynamic quantization may further optimize the balance between accuracy and computational efficiency. Integration with deep-learning pipelines as a preprocessing stage could improve noise robustness for computer-aided diagnostic systems while maintaining low computational overhead. Additionally, implementing the framework on certified point-of-care or mobile imaging devices and conducting prospective clinical validation would establish practical utility. Further exploration of pipelined, parallelized, and memory-reuse architectures on FPGA, ASIC, or embedded GPU platforms could enable sub-millisecond latency for high-resolution streaming, while energy-aware scheduling, dynamic voltage/frequency scaling, and power gating could minimize energy consumption in battery-operated environments. Collectively, these strategies aim to position the integer-only FLBMF as a robust, scalable, and clinically validated component of next-generation intelligent healthcare imaging systems.

## Data Availability

The mammogram images used in this study are publicly available from The Cancer Imaging Archive (TCIA) under the Curated Breast Imaging Subset of the Digital Database for Screening Mammography (CBIS-DDSM) collection and can be accessed at: [https://www.cancerimagingarchive.net/collection/cbis-ddsm/](https:/www.cancerimagingarchive.net/collection/cbis-ddsm) The dataset is referenced by DOI: https://doi.org/10.7937/K9/TCIA.2016.7O02S9CY.
